# Future HIV epidemic trajectories in South Africa and projected long-term consequences of reductions in general population HIV testing: a mathematical modelling study

**DOI:** 10.1016/S2468-2667(24)00020-3

**Published:** 2024-03-27

**Authors:** Stefan P Rautenbach, Lilith K Whittles, Gesine Meyer-Rath, Lise Jamieson, Thato Chidarikire, Leigh F Johnson, Jeffrey W Imai-Eaton

**Affiliations:** a MRC Centre for Global Infectious Disease Analysis, School of Public Health, Imperial College London, London, UK; b Health Economics and Epidemiology Research Office, Faculty of Health Sciences, University of the Witwatersrand, Johannesburg, South Africa; c Department of Global Health, School of Public Health, Boston University, Boston, MA, USA; d South African Department of Science and Innovation and National Research Foundation Centre of Excellence in Epidemiological Modelling and Analysis, Stellenbosch University, Stellenbosch, South Africa; e HIV and AIDS and STI Unit, National Department of Health, Johannesburg, South Africa; f Centre for Infectious Disease Epidemiology and Research, School of Public Health, University of Cape Town, Cape Town, South Africa; g Center for Communicable Disease Dynamics, Department of Epidemiology, Harvard TH Chan School of Public Health, Boston, MA, USA

## Abstract

**Background:**

After successful intensive interventions to rapidly increase HIV awareness, coverage of antiretroviral therapy (ART), and viral suppression, HIV programmes in eastern and southern Africa are considering scaling back of some interventions, such as widespread general population HIV testing. We aimed to model whether scaling back of general population HIV testing in South Africa could result in a resurgence of the HIV epidemic or substantial slowing of declines in HIV incidence, resulting in increased long-term ART.

**Methods:**

In this modelling study, we used the Thembisa 4.5 model (a deterministic compartmental model of HIV transmission in South Africa) to project the South African HIV epidemic to 2100 assuming the continuation of 2022 epidemiological conditions and HIV programme implementation. We assessed how implementing reductions in general population HIV testing services in 2025 (while maintaining antenatal, symptom-based, and risk-based testing modalities and other HIV prevention services at 2022 levels) would affect HIV incidence and prevalence among people aged 15–49 years, the year in which incidence would reach one per 1000 people aged 15–49 years (the threshold for virtual elimination of HIV), and associated costs, as well as numbers of additional new HIV infections and AIDS-related deaths. We also modelled the effects of delaying reductions in general population testing services by 5-year increments. Additionally, we modelled the potential effects of reductions in general population testing services in combination with increases or decreases in ART interruption rates (ie, the annual rate at which people who are on ART discontinue ART) and condom usage in 2025–35.

**Findings:**

If general population HIV testing services and the HIV risk environment of 2022 were maintained, we projected that HIV incidence would steadily decline from 4·95 (95% CI 4·40–5·34) per 1000 population in 2025 to 0·14 (0·05–0·31) per 1000 in 2100, and that the so-called virtual elimination threshold of less than one new infection per 1000 population per year would be reached in 2055 (95% CI 2051–2060). Scaling back of general population HIV testing services by 25%, 50%, or 75% in 2025 delayed time to reaching the virtual elimination threshold by 5, 13, or 35 years, respectively, whereas complete cessation of general population testing would result in the threshold not being attained by 2100. Although the incidence of HIV continued to fall when general HIV testing services were reduced, our modelling suggested that, with reductions of between 25% and 100%, between 396 000 (95% CI 299 000–474 000) and 2·50 million (1·97 million–2·98 million) additional HIV infections and between 115 000 (94 000–135 000) and 795 000 (670 000–926 000) additional AIDS-related deaths would occur between 2025 and 2075, depending on the extent of reduction in testing. Delaying reductions in general population HIV testing services for 5–25 years mitigated some of these effects. HIV testing accounted for only 5% of total programmatic costs at baseline; reducing testing moderately reduced short-term total annual costs, but increased annual costs after 25 years. Increases in ART interruption and reductions in condom usage were projected to slow the decline in incidence and increase the coverage of general HIV testing services required to control transmission but did not cause rapid resurgence in HIV infections.

**Interpretation:**

Our modelling suggests that scaling back of general population HIV testing would not result in a resurgence of HIV infections, but would delay attainment of incidence-reduction targets and result in long-term increases in HIV infections, AIDS-related deaths, and costs (via increased need for ART provision). HIV programmes need to balance short-term potential resource savings with long-term epidemic control objectives.

**Funding:**

Bill & Melinda Gates Foundation.

## Introduction

Over the past decade, HIV programmes in eastern and southern Africa have substantially expanded HIV testing, access to antiretroviral therapy (ART), and other interventions to meet UNAIDS 95-95-95 targets^1^, ^2^ (ie, that 95% of people living with HIV know their HIV status, that 95% of people who know that they have HIV are on ART, and that 95% of people who are on treatment are virally suppressed). These efforts aimed to rapidly increase viral suppression at the population level and reduce the number of new HIV infections and AIDS-related deaths as part of commitments to end AIDS as a public health threat by 2030.^1^, ^3^, ^4^ These intensive programmes have helped to increase the proportion of people with HIV with viral suppression in the region from 45% in 2015 to 77% in 2022, and five countries have already attained the 95-95-95 targets.^2^

**Research in context**

**Evidence before this study**

High rates of HIV testing and early eligibility for treatment have increased treatment coverage and, in combination with primary prevention, accelerated the steadily declining incidence of new HIV infections in eastern and southern Africa. National HIV programmes are seeking long-term strategies to sustain declines in, and eventually eliminate, new infections. With coverage of antiretroviral therapy (ART) now high, many countries in the region are beginning to scale back large HIV testing programmes that were implemented to rapidly reach the UNAIDS 90-90-90 targets, which then became the 95-95-95 targets (ie, that 95% of people living with HIV know their HIV status, 95% of people who know that they have HIV will be on ART, and 95% of people who are on treatment will be virally suppressed). However, how reductions in testing programmes or future changes in HIV treatment or sexual risk behaviours could affect the long-term incidence of new HIV infections is unknown. We searched PubMed with the terms “HIV” [Title] AND “Africa” [All Fields] AND “incidence” [All Fields] AND (“model*” [Title/Abstract] OR “project*” [Title/Abstract] OR “forecast*” [Title/Abstract]) AND (“elimination” [All Fields] OR “epidemic control” [All Fields]) for studies in which mathematical models were used to project long-term HIV incidence in eastern and southern Africa published in any language from database inception to June 27, 2023. We found no studies on the effect of reductions in HIV testing on the long-term incidence of HIV. Three studies projected long-term HIV incidence in South Africa or Eswatini using current or altered epidemic and programmatic conditions. One study, in which the incidence of HIV was projected from 2019 to 2039 in South Africa, suggested that a decline in incidence to one new infection per 1000 susceptible HIV-negative population (aa proposed threshold for so-called virtual elimination of HIV) was unlikely to be attained under any conditions before 2040. Another study projected incidence from 2013 to 2063 in South Africa. Its findings suggested that an incidence of one new infection per 1000 population per year would not be attained if the testing levels and treatment guidelines at the time (ie, prescription of ART only to people with <350 CD4 cells μL) were maintained, but would be possible if universal testing (ie, annual testing of the entire population) and subsequent immediate treatment were introduced or if condoms were used in all sex acts. In the third study, in which HIV incidence in Eswatini was projected between 2016 and 2050, modelling suggested that an incidence of one new infection per 1000 population per year would not be attained under any conditions by 2050.

**Added value of this study**

In this modelling study, we assessed the potential effects of sustaining or scaling back general population HIV testing in South Africa on long-term HIV incidence, additional infections, and AIDS-related deaths from 2025 to 2100. When sustaining programmatic and epidemic conditions, the incidence of new HIV infections was projected to continue to decrease, reaching an incidence of less than one new infection per 1000 population per year and an incidence of 0·14 (95% CI 0·05–0·31) new infections per 1000 people in 2100. Declining incidence was robust to reduced HIV testing, treatment retention, and condom usage. Large changes in any of these factors individually did not result in rapid surges in new infections. Even with large reductions in general population HIV testing services, the incidence of HIV continued to decrease, but this slowed pace resulted in more new HIV infections, AIDS-related deaths, and need for treatment. Declines in HIV incidence were reversed only in extreme scenarios in which condom use decreased in combination with a cessation of general population HIV testing services. Reducing testing modestly reduced short-term total annual costs, but increased annual costs after 25 years due to an increase in people requiring ART.

**Implications of all the available evidence**

In settings that have achieved high treatment coverage and viral suppression, managed reductions of general population HIV testing services are unlikely to produce a surge in new HIV infections. However, attainment of incidence reduction targets might be delayed, together with long-term increases in HIV infections, AIDS-related deaths, and costs (via increased need for ART provision). Increases in treatment interruption and sexual risk behaviour alongside reductions in general population testing could result in more new infections and deaths and increased expenditure on HIV care and treatment over time. HIV programmes face policy decisions about the opportunity to save or reallocate short-term resources by reducing general population testing services, which needs to be balanced with potential slowing of progress towards reducing new HIV infections and controlling the epidemic.

Many HIV programmes are now considering how to sustain high levels of viral suppression and low and declining numbers of new infections in the future. One consideration is whether, when, and by how much to scale back resource-intensive vertical programmes without risking a potential resurgence of HIV infections or deaths or slowing of trends in declining incidence (thereby increasing long-term treatment costs). Since around 2018, several countries, including Lesotho, Uganda, and Kenya, have reduced general population HIV testing services substantially. Long-term programme management decisions should be robust to uncertainty about future incidence dynamics. Potential sources of uncertainty include observable programme outcomes to which strategies could respond dynamically—eg, improvement or deterioration in ART retention (ie, remaining enrolled in ART programmes) and viral suppression—or harder-to-monitor determinants, such as changes in population risk behaviours (eg, increased numbers of sexual partners, decreased condom use).

In South Africa, where 7·8 million people have HIV (almost one in five adults),^5^ 14 million HIV tests are done yearly—a number that is projected to increase to 20 million yearly by 2039 if testing trends continue.^6^ New HIV infections in South Africa have declined since 1997, including by 58% since 2010.^5^ Declines in the number of new infections were initially largely attributed to increased condom usage^7^, ^8^ and, since around 2010, to the scaling up of ART coverage via extensive HIV testing and expanded eligibility, along with other preventive interventions such as voluntary medical male circumcision.^8^, ^9^ General population HIV testing (as opposed to risk-targeted, symptomatic, or antenatal testing) accounts for most HIV tests in South Africa,^6^ and could be scaled back as the number of untreated people with HIV decreases.

We used a mathematical model of HIV transmission in South Africa^10^ to simulate long-term epidemic trajectories up to 2100 on the basis of current trends (as of 2022) in testing, ART linkage and interruption, and uptake of other HIV prevention interventions. We assessed how managed reductions in general population HIV testing affected HIV infections, AIDS-related deaths, and HIV programme costs. We also considered the potential epidemiological effects of changes in ART retention or condom use under scenarios in which general population testing had been scaled back.

## Methods

### Study design, setting, and model overview

In this mathematical modelling study, we used Thembisa, a deterministic compartmental model of HIV transmission and demographics in South Africa, which is used to generate national HIV estimates and simulate alternative HIV policy scenarios, including estimates reported by UNAIDS.^8^, ^10^ The model is calibrated to national data using a Bayesian approach to HIV prevalence, mortality, sexual partnership and risk behaviour, programme testing, treatment, and prevention provision.^10^ We used the Thembisa 4.5 model, which was updated in 2022 (an updated version, Thembisa 4.6, has been released since this paper was submitted).

Model details and calibration have been described elsewhere.^10^ Briefly, the population was stratified by demographic groups (ie, by age and sex), behavioural risk (ie, by sexual experience [ever having had sex], marital status, propensity for partner concurrency, and engagement in sex work), and HIV status. The HIV-positive population was further stratified by infection stage, HIV testing history, and ART use. The model assumes that HIV epidemic in South Africa was initialised in 1985. HIV transmission in the model depends on sexual mixing and the distribution of the HIV-positive population according to infection stage and ART use (reflecting variation in viral load), awareness of HIV-positive status, use of preventive methods (eg, condoms or pre-exposure prophylaxis [PrEP]), and circumcision status for HIV-negative men.

HIV diagnosis and linkage to ART occurs via seven routes in the model: testing of pregnant women at antenatal clinics, testing prompted by opportunistic infections, testing of HIV-exposed infants, testing of sexual partners of people with HIV through passive partner notification, self-testing, testing among people taking PrEP (which is recommended four times per year in South African guidelines), and general population HIV testing. General population testing includes provider-initiated, insurer-initiated, or self-initiated HIV testing of asymptomatic, non-pregnant adults.

### Mathematical modelling

We used Thembisa to project the South African HIV epidemic from 2025 to 2100 assuming the continuation of 2022 demographic trends and previously inferred trends in condom use, ART initiation and retention, and HIV testing (appendix pp 2–3), using 1000 calibrated model parameter sets to incorporate parametric uncertainty. In the model, whereas demographic factors (eg, fertility, non-AIDS mortality, migration) continue to change towards a specified equilibrium level according to default demographic projection assumptions, HIV testing, ART initiation, and retention rates are held constant at 2022 levels. Condom use rates are constant within partnership types, but total condom use changes over time slightly because of changes in composition of partnership type as a result of historical changes in marital rates. HIV self-testing rates were low before 2021 and assumed to be zero from 2022 onwards (because rates were judged too low to be relevant). Uptake of other prevention interventions, including condom use, voluntary medical male circumcision, and PrEP use, among key populations and populations at high risk was assumed to continue at 2022 levels. Our long-term projections did not incorporate all factors that could affect the epidemic, such as technological advancements (eg, HIV vaccines, functional cures), changes in uptake of other prevention methods (eg, voluntary medical male circumcision, PrEP), or unforeseen events or structural changes. Our projections therefore reflect scenarios based on current trends but not future predictions.

To enable comparisons, we simulated a status quo scenario in which the programme levels and risk environment of 2022 were maintained up to 2100. To assess the potential effect of future reductions in general population HIV testing, we simulated how the epidemic would develop assuming reductions of between 0% and 100% in general population testing compared with the 2022 rate (appendix p 4). Reductions in general population testing were implemented over 2 years (with steady reduction in the testing rate each simulation month over the 2-year period) and then maintained at the lower rate until 2100. Antenatal, symptom-based, and passive partner notification testing were maintained at 2022 rates. To assess the effects of delaying reductions in testing, reductions in testing in our model were implemented every 5 years between 2025 and 2050.

We assessed how reductions in HIV testing interacted with other potential positive or negative epidemiological changes by varying the rate of ART interruption (ie, of discontinuation of daily ART treatment) and condom use (as a percentage of sex acts in which a condom is used) over 10 years (from 2025 to 2035). We varied ART interruption in scenarios ranging from a 14% annual reduction in the proportion of people with ART interruption to a 14% annual increase in the proportion of people with ART interruption relative to the 2022 baseline (a reasonable range to encompass extreme but plausible future changes; apppendix p 4), corresponding to ART coverage in 2035 of 50–92% (compared with 77% under the status quo scenario). Similarly, we modelled future changes in sexual risk by varying the odds of condom use, from a 14% reduction in use to a 14% increase in use between 2025 and 2035 relative to the 2022 baseline to encompass a wide but reasonable range for future potential change (appendix p 4). Modelled scenarios thus considered how condom use of between 14% and 58% in 2035 would affect the HIV epidemic (compared with condom use of 34% under the status quo scenario).

Model outputs were annual HIV incidence per 1000 susceptible HIV-negative population aged 15–49 years (a commonly used age range for reporting HIV rate indicators in the adult population of reproductive age), prevalence of HIV among people aged 15–49 years, HIV testing volume, treatment coverage, numbers of new infections, and AIDS-related deaths among people aged 15 years or older. We calculated the time to reach an HIV incidence of less than one per 1000 people aged 15–49 years (a proposed threshold for virtual HIV elimination).^11^ We also calculated the additional HIV infections and AIDS-related deaths that would occur over a 50-year period from when general population HIV testing was reduced (ie, in 2075 in analyses where HIV testing was reduced in 2025) because use of cumulative outcomes over 50 years enabled comparison over a consistent time horizon for interventions implemented at intervals between 2025 and 2050.

We estimated annual and cumulative HIV testing and treatment programme costs of the alternative scenarios by applying unit costs to epidemiological outcomes from the Thembisa model.^6^, ^12^ Costs were estimated from the perspective of the provider (South African Government) and presented in 2023 US$. Costs were included for HIV testing (non-antenatal care testing $3·63 per HIV-negative test and $5·20 per HIV-positive test; antenatal care testing $3·16 per HIV-negative test and $4·72 per HIV-positive test), ART ($272 in the first year then $171 per subsequent year or $300 per year for second-line treatment), and inpatient and palliative care (ranging from $37 to $402 per patient-year depending on ART and CD4 cell count; appendix p 6).^6^, ^12^ Other programme costs, such as HIV prevention costs, were excluded. We calculated the incremental difference in annual testing and treatment costs over time compared with the status quo and cumulative costs after 5, 10, 25, and 50 years discounted at 0%, 3%, and 6% per year (a conventional range of discount rates applied to health economic analyses). Fixed values were used for all cost inputs; uncertainty ranges for cost results reflect uncertainty in epidemiological outcomes only.

The Thembisa model was implemented in C++14. Model results were processed and analysed in R (version 4.2.2).

### Role of the funding source

The funder of the study had no role in study design, data collection, data analysis, data interpretation, or writing of the report.

## Results

**Figure 1:**
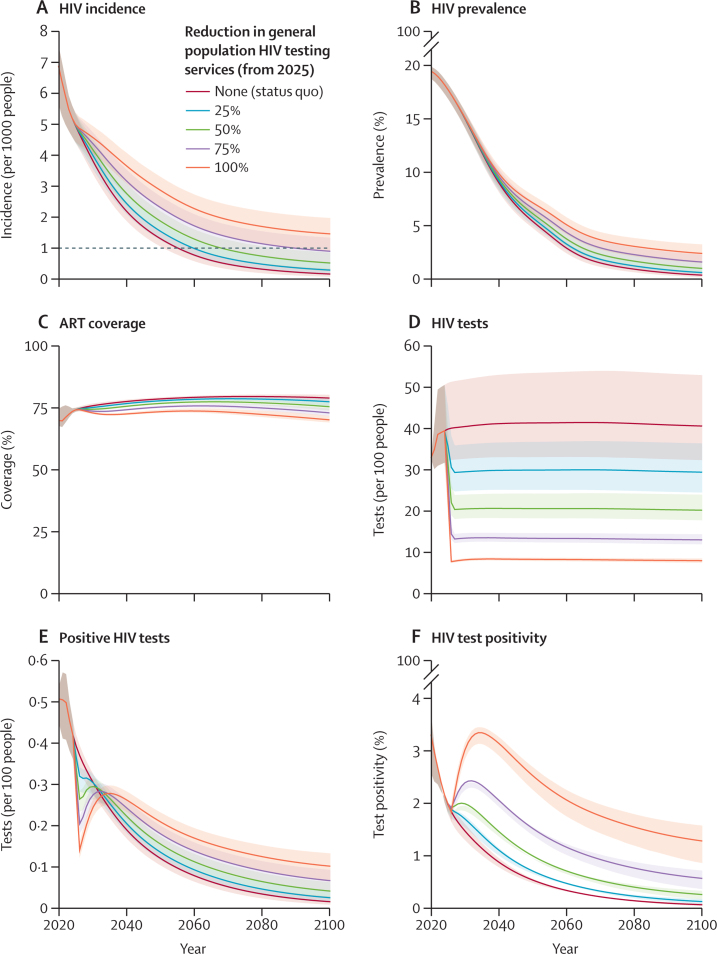
Projected effects of reducing general population HIV testing services in South Africa in 2025 on HIV incidence and programmatic outcomes (A) Projected HIV incidence per 1000 HIV-negative people aged 15–49 years per year. The dotted line represents an incidence of one new infection per 1000 people per year (the threshold for so-called virtual elimination). (B) Projected HIV prevalence among people aged 15–49 years. (C) Projected ART coverage among people aged 15 years or older with HIV. (D) Projected number of HIV tests per 100 people older than 15 years per year. (E) Projected number of positive HIV tests per 100 people aged 15 years or older. (F) Projected proportion of HIV tests taken by people aged 15 years or older that were positive. In A–F, trend lines represent posterior means and shaded areas are 95% CIs. ART=antiretroviral therapy.

In the status quo scenario, the incidence of HIV in people aged 15–49 years in South Africa was projected to continue to decline steadily from 4·95 (95% CI 4·40–5·34) per 1000 population in 2025, to 0·14 (0·05–0·31) per 1000 population in 2100 (figure 1A), falling below one case per 1000 population per year in 2055 (95% CI 2051–2060). The prevalence of HIV among people aged 15–49 years was projected to decrease from 17·7% (95% CI 16·8–18·2) in 2025 to 4·1% (3·6–4·7) in 2055 and 0·38% (0·19–0·70) by 2100 (figure 1B). We projected that maintaining current testing rates would increase the testing volume from 17·6 million tests (14·2 million–22·5 million) in 2025 to 24·3 million (19·4 million–31·5 million) by 2055 and would result in around 78% (77–80) of people aged 15 years or older with HIV being on ART (figure 1C; appendix p 20). Under this scenario, we projected 3·88 million (3·37 million–4·38 million) new HIV infections and 1·75 million (1·62 million–1·88 million) AIDS-related deaths between 2025 and 2075 among people aged 15 years or older. These projections were similar to the number of new HIV infections that occurred in South Africa in the 13 years between 2009 and 2022 and of AIDS-related deaths in the 16 years between 2006 and 2022.^10^

In all scenarios in which general population HIV testing services were reduced from 2025, the incidence of new HIV infections was projected to continue to decline, but the rate of decline slowed compared with the status quo scenario (figure 1A). Fully ceasing general population HIV testing (ie, retaining only antenatal, symptom-based, and passive partner testing) from 2025 reduced testing volume from around 40 tests per 100 adults per year to eight tests per 100 adults per year (figure 1D). The number of diagnoses was projected to initially decrease commensurately with testing reductions, but then increase to a higher number of diagnoses than in the status quo scenario from 2035 due to later diagnosis through antenatal and symptom-based testing (figure 1E). Reducing general population HIV testing services by 25% from 2025 increased projected HIV incidence in 2100 to 0·27 (95% CI 0·11–0·53) per 1000 population aged 15–49 years, compared with 0·50 (0·24–0·84) for a 50% reduction, 0·88 (0·47–1·32) for a 75% reduction, and 1·44 (0·86–1·96) for a 100% reduction. Reducing general population HIV testing by 25%, 50%, and 75% was projected to delay achievement of the target of incidence below 1 case per 1000 people per year by 5, 13, and 35 years, respectively, beyond 2055 (when we projected the threshold would be reached under the status quo scenario); this threshold was not reached before 2100 when general HIV testing was reduced by more than 80% (figure 1A). HIV prevalence in 2100 was also commensurately higher when general population testing was reduced from 2025 compared with the status quo scenario (figure 1B).

ART coverage in 2100 was 78% (95% CI 77–80) in the status quo scenario, compared with 77% (75–79) when general testing was scaled back by 25%, 75% (74–77) when testing was scaled back by 50%, 73% (72–75) when testing was scaled back by 75%, and 70% (69–72) when testing was scaled back by 100% (figure 1C). This reduction in ART coverage secondary to reductions in general HIV testing was projected to result in additional HIV infections between 2025 and 2075: 396 000 (299 000–474 000) with a 25% reduction in general population testing, 926 000 (718 000–1·11 million) with a 50% reduction, 1·63 million (1·27 million–1·96 million) with a 75% reduction, and 2·50 million (1·97 million–2·98 million) with a 100% reduction in general population testing from 2025 (appendix p 20). The number of AIDS-related deaths between 2025 and 2075 was projected to increase by between 115 000 (94 000–135 000) when general testing was scaled back by 25% and 795 000 (670 000–926 000) when testing was scaled back by 100% from 2025. Despite lower proportional HIV coverage, reductions in general population HIV testing in 2025 were projected to increase the number of adults receiving ART in 2075 (because of higher numbers of infections) from 2·66 million (2·34 million–2·96 million) in the status quo scenario to 2·83 million (2·48 million–3·14 million) when general population testing was reduced by 25%, or to 3·58 million (3·05 million–3·98 million) when general population HIV testing was reduced by 100% (appendix p 20).

**Figure 2:**
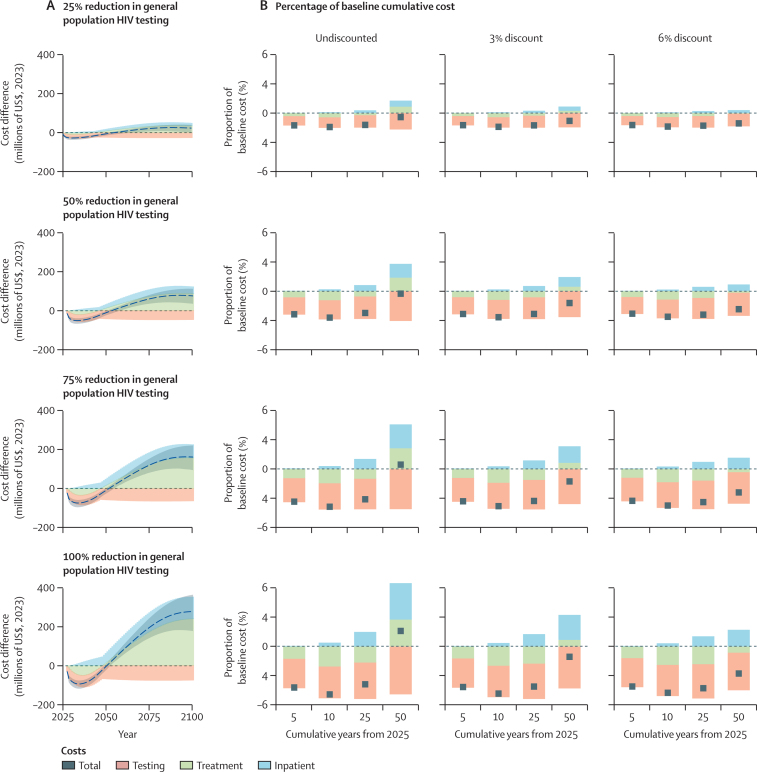
Projected effects of reducing general population HIV testing services in South Africa in 2025 on HIV testing and treatment costs (A) Incremental difference in undiscounted annual programme costs, 2025–2100, when reducing general population HIV testing services in 2025 compared with the status quo scenario (baseline). The dashed line represents the posterior mean change in total cost summed for all three components and shaded areas are 95% CIs for the change in total costs. (B) Incremental difference in cumulative programme costs as a proportion of the cumulative status quo (baseline) cost after reducing general population HIV testing services in 2025. Cumulative costs are reported for 0% (undiscounted), 3%, and 6% discounts per year. The grey squares represent changes in total cost summed for all three components.

In the status quo scenario, HIV testing accounted for $676 million (95% CI 560 million–847 million) of the $14·5 billion HIV testing and treatment programme cost for 2025–34, corresponding to 4·7% (95% CI 4·0–5·7; a 3% discount per year was applied in the status quo scenario; appendix p 19). Total annual costs (undiscounted) were projected to peak around 2034 and to decline thereafter in all scenarios (appendix p 19), coinciding with decreasing numbers of people with HIV—and thus decreasing numbers of people taking ART (appendix p 20). Reducing general population HIV testing services by between 25% and 100% was projected to reduce total costs of HIV testing and treatment from 2025 to 2034 (3% discount per year) by between 1·4% ($203 million [95% CI 153 million–274 million]) and 4·8% ($700 million [560 million–889 million]; figure 2B). Most of this reduction was from reduced testing costs, between $154 million (108 million–218 million) and $468 million (357 million–629 million) lower than in the status quo scenario (with a 25% and 100% reduction in general testing, respectively), accounting for 76% to 67% of total reductions in cost. Some savings were related to reduced ART costs (between $61 million [50 million–71 million] and $284 million [242 million–324 million] lower than in the status quo scenario), accounting for 30% to 41% of the total reduction when general testing was reduced by 25% and 100%, respectively, as a result of fewer people being diagnosed with HIV and beginning ART (appendix p 20).

Reduced general population testing was projected to lower total annual costs of HIV testing, care, and treatment programmes compared with the status quo scenario between 2025 and 2050, but annual costs were higher than under the status quo scenario after 2050 (figure 2A) as a result of a projected increase in new infections resulting in more people requiring ART and greater care costs for people with HIV (appendix p 19). Any degree of reduction in general population testing was projected to result in increased inpatient costs compared with the status quo scenario as a result of a projected increase in people with HIV due to increased infections, later diagnosis, and more AIDS-related deaths (figure 2A). Over 50 years, reducing general population HIV testing services by 50% was projected to save $427 million (237 million–828 million), corresponding to a 1·2% decrease in the total cumulative cost in the status quo scenario over 50 years (which was estimated to be $38·8 billion [36·5 billion–40·1 billion]; 3% discount per year; figure 2B). Cessation of general population HIV testing in 2025 decreased 50-year cumulative total costs of HIV testing, care, and treatment programmes by $406 million (57 million–915 million), corresponding to a 1·0% decrease in the total cumulative cost in the status quo scenario over 50 years. Modest savings were made when future costs were discounted by 3% per annum, whereas undiscounted cumulative costs were 2·0% higher over 50 years than undiscounted costs in the status quo scenario (figure 2B). When future costs were discounted by 6% per annum, 50-year cumulative costs were 2·8% lower than in the status quo scenario because savings occurred in the short-term while increased treatment costs were incurred three decades in the future (figure 2B).

**Figure 3:**
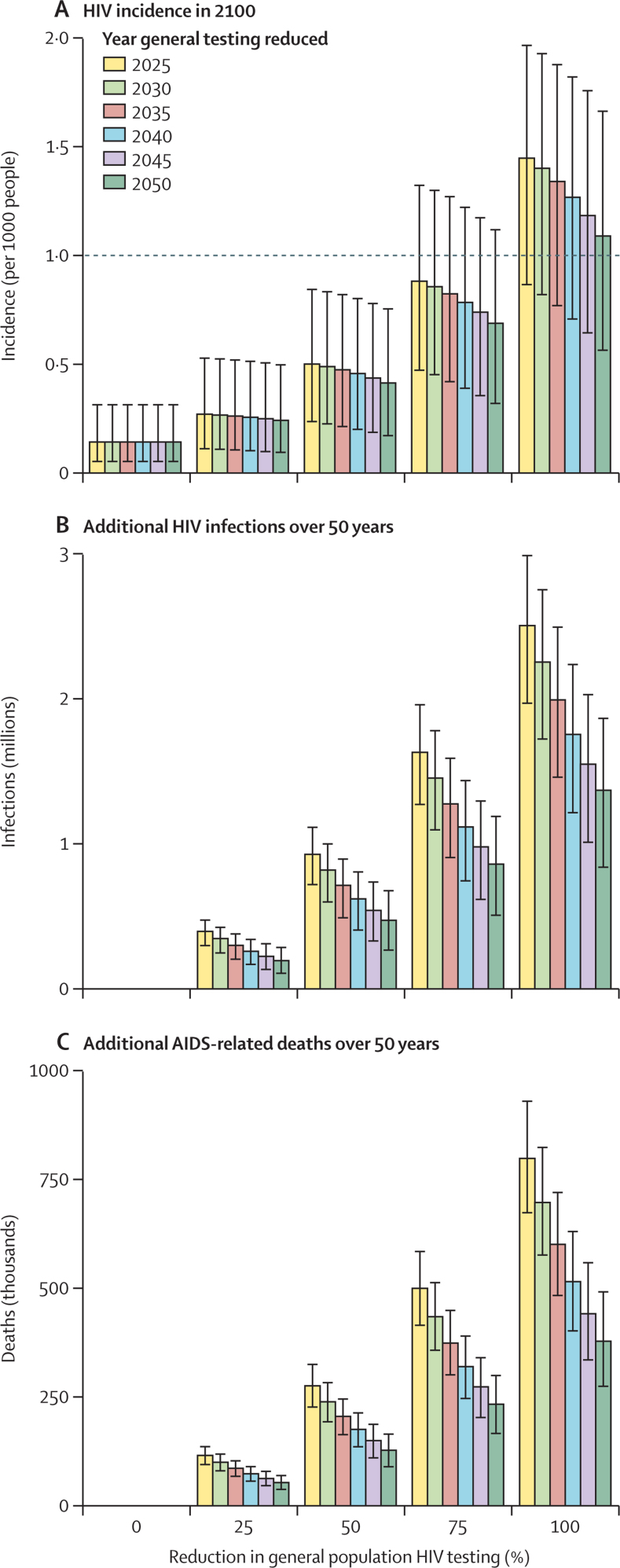
Projected HIV incidence in 2100 and cumulative epidemiological effects over 50 years after reducing general population HIV testing services, by year in which testing was reduced (A) Projected HIV incidence per 1000 people aged 15–49 years in 2100. The dotted line represents an incidence of one new HIV infection per 1000 people per year (the threshold for so-called virtual elimination). (B) Projected additional HIV infections in people aged 15 years or older over 50 years. (C) Additional AIDS-related deaths over 50 years. Data are posterior means; error bars represent 95% CIs.

Delaying reductions in general population HIV testing services beyond 2025 was projected to modestly affect long-term incidence in 2100 (figure 3A), but was projected to have a larger effect on the cumulative numbers of additional infections and deaths (figure 3B, 3C). For example, a 50% reduction in general population testing from 2050 resulted in a projected incidence of HIV in 2100 of 0·41 (95% CI 0·17–0·75) per 1000 people aged 15–49 years, compared with an incidence of 0·50 (0·24–0·84) per 1000 when testing was reduced by that proportion from 2025. However, delaying a 50% reduction in general population testing from 2025 to 2030 decreased the number of new HIV infections from 926 000 (95% CI 718 000–1·11 million) to 818 000 (599 000–998 000) and decreased AIDS-related deaths from 274 000 (225 000–323 000) to 237 000 (192 000–281 000) over 50 years. Although delaying a 50% reduction in general population HIV testing services until 2050 decreased expected new HIV infections and AIDS-related deaths, this reduction was still associated with a projected additional 472 000 (268 000–676 000) infections and 126 000 (89 000–163 000) deaths between 2050 and 2100 compared with the status quo scenario.

**Figure 4:**
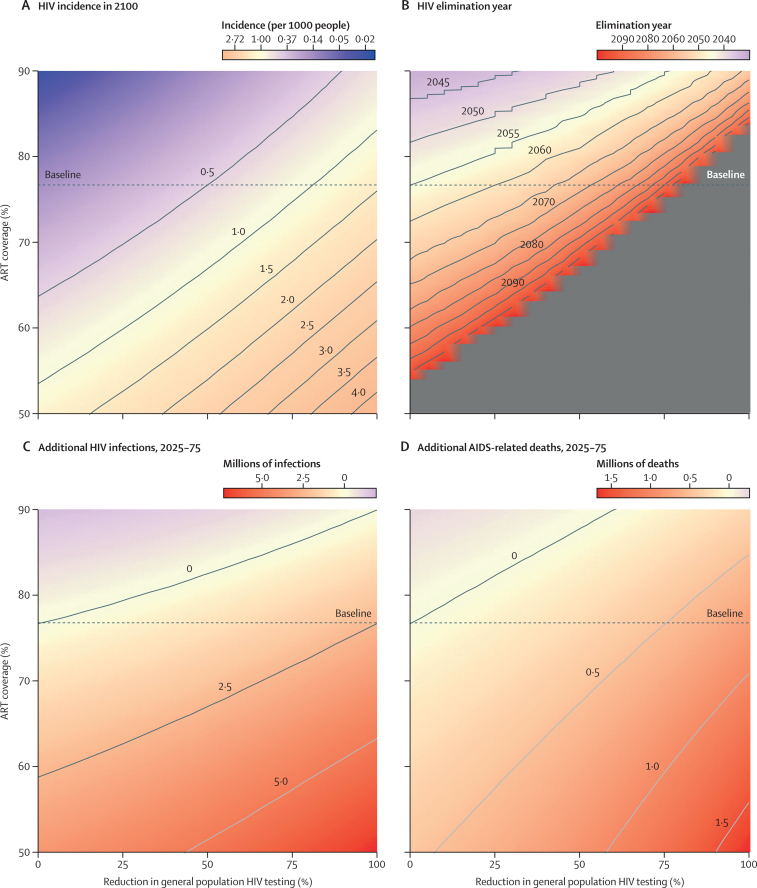
Heatmaps of the effects of reductions in general population HIV testing from 2025 and changes in ART coverage (A) Projected HIV incidence per 1000 people aged 15–49 years in 2100. (B) Year in which HIV incidence is projected to fall below one new infection per 1000 people aged 15–49 years (the threshold for so-called virtual elimination of HIV). The grey area represents combinations of reduced general population testing and ART coverage that result in the virtual elimination threshold not being met by 2100. (C) Projected additional HIV infections over 50 years (ie, between 2025 and 2075). (D) Projected additional AIDS-related deaths over 50 years (ie, between 2025 and 2075). For all heatmaps, modelled changes in ART coverage were implemented over 10 years from 2025; the y-axis shows ART coverage in 2035. In all heatmaps, the dotted baseline represents the ART coverage in 2035 in the status quo scenario. ART=antiretroviral therapy.

Increasing the proportion of people who interrupted ART between 2025 and 2035 was not projected to result in rapidly rebounding new HIV infections or AIDS-related deaths (appendix p 16), even when combined with reductions in HIV general population testing (appendix p 13). However, increased treatment interruption was projected to result in increased long-term incidence, longer time to an incidence of less than one case per 1000 people aged 15–49 years per year, and more cumulative infections and AIDS-related deaths compared with the status quo scenario (figure 4). In the most extreme scenario, in which general HIV testing ceased from 2025 and ART coverage fell to 50% in 2035, HIV incidence in 2100 remained high (4·32 cases [95% CI 2·99–5·36] per 1000 population; figure 4A), similar to the incidence in 2025 (4·95 cases [4·40–5·34] per 1000). Conversely, improved ART retention was projected to accelerate reductions in incidence or enable increased or earlier reductions in general population testing to attain the same epidemiological effects. If ART coverage increased to 80%, an incidence of less than one case of HIV per 1000 population per year could be attained by 2055 with up to a 25% reduction in gereral HIV testing; if ART coverage increased to 90% this incidence could be reached even with a 75% reduction in general HIV testing (figure 4B). Overall, for every 1% increase in ART coverage in 2035 (up to 90%), general population HIV testing could be reduced by 4·5% in 2025 without increasing expected deaths or infections (figure 4C, D).

**Figure 5:**
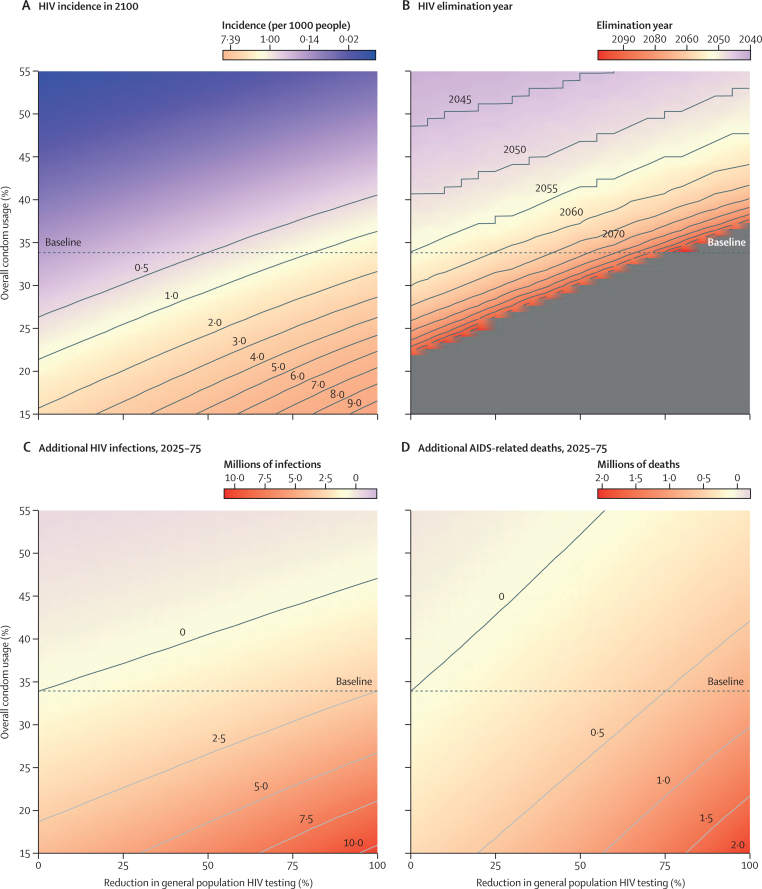
Heatmaps of the effects of reductions in general HIV testing from 2025 and changes in condom use (A) Projected HIV incidence per 1000 people aged 15–49 years in 2100. (B) Year in which HIV incidence is projected to fall below one new infection per 1000 people aged 15–49 years (the threshold for so-called virtual elimination of HIV). The grey area represents combinations of reduced general testing and condom use that result in the virtual elimination threshold not being met by 2100. (C) Projected additional HIV infections over 50 years (ie, between 2025 and 2075). (D) Projected additional AIDS-related deaths over 50 years (ie, between 2025 and 2075). For all heatmaps, modelled changes in condom use were implemented over 10 years from 2025; the y-axis shows the proportion of sexual acts in which condoms were used in 2035. In all heatmaps, the dotted baseline represents the proportion of sexual acts in which condoms were used in 2035 in the status quo scenario.

The epidemic trajectory was more sensitive to future changes in condom use than to future changes in ART interruption. We projected that declines in HIV incidence would be reversed by 2035 if the proportion of sexual acts in which condoms were used fell from 34% to 14% and general population HIV testing services were reduced by more than 75% (figure 5A; appendix p 18). Reducing the porportion of sexual acts in which condoms were used from 34% to 26% delayed reaching an incidence of less than one new HIV infection per 1000 people aged 15–49 years per year by 14 years compared with the status quo, to 2069 (95% CI 2060–2093), and, if condoms were used in only 21% of sex acts from 2035 onwards, reaching this incidence before 2100 was projected to be unlikely (figure 5B).

In a scenario in which current testing rates were maintained but condom use fell to 14%, an additional 3·66 million (95% CI 2·87 million–4·52 million) infections and 346 000 (262 000–442 000) AIDS-related deaths were projected between 2025 and 2075 compared with the status quo scenario. Cessation of general population HIV testing services in combination with condom use falling to 14% by 2035 led to a projected 10·9 million (10·0 million–11·8 million) more infections and 2·04 million (1·83 million–2·21 million) more AIDS-related deaths by 2075 compared with the status quo scenario. If general population HIV testing ceased in 2025, condom use would need to increase to more than 46% to maintain the same number of new HIV infections as in the status quo scenario (figure 5C). For every 1% increase in condom use in 2035 (up to 56%), general HIV population testing services could be reduced by 2·7% in 2025 without additional infections or deaths (figure 5C, D).

## Discussion

In this modelling study, we projected that, if general HIV testing services and the HIV risk environment remained at 2022 levels, HIV incidence in South Africa would continue to steadily decline for several decades, falling to an incidence of less than one new infection per 1000 people aged 15–49 years per year around 2055. We projected that the proportion of adults aged 15–49 years living with HIV would fall from around one in five to less than one in 100 by 2100. Managed reductions in HIV testing did not lead to rapid increases in new infections, but were projected to slow the rate of decline in incidence and increase the future number of infections, AIDS-related deaths, and people requiring lifelong ART. HIV testing contributed only around 5% of total HIV testing and treatment costs in the status quo scenario. Reducing testing could enable some resources to be allocated to other short-term health-system priorities, but would result in slowed progress towards epidemic control and associated increases in long-term HIV programme costs.

Other countries in eastern and southern Africa with higher ART coverage than South Africa have already begun reducing large-scale general population testing while promoting targeted testing approaches, partly as a result of shifts in donor resources from the US President's Emergency Plan for AIDS Relief (PEPFAR).^13^ Our findings suggest that these programme shifts are unlikely to lead to reversed progress within the next 10–20 years, but also highlight the potential long-term costs of slowed reductions in incidence. In our projections, cumulative HIV programme costs and cost uncertainty were primarily determined by future numbers of people acquiring HIV who will require lifelong ART. Maintaining current HIV testing rates for 5–25 years longer mitigated some of the effects of reducing HIV testing services and might be a good strategy, even in contexts with low and falling numbers of untreated people with HIV, given the low cost of testing compared with lifelong HIV care. HIV testing approaches could be increasingly integrated into general disease screening programmes, supporting commitments to universal health coverage.^14^, ^15^ More flexible and targeted HIV testing services for those at increased risk of infection could be another strategy to mitigate negative effects of reducing general population testing services,^16^ but evidence for risk-screening tools suggests that the effectiveness of such strategies could be restricted.^17^

Our primary projections assumed that retention on ART, viral suppression, uptake of other HIV preventive approaches (including voluntary medical male circumcision, PrEP, and condom use), and sexual risk behaviour are sustained at 2022 levels. In scenarios representing an increase in ART interruption or increased sexual risk in the next decade but not catastrophic discontinuation (an unmanaged large-scale disruption of ART service provision—eg, treatment becomes no longer available because supply chains are shut down, as was imagined and modelled as a worst-case scenario during the COVID-19 pandemic), the combination of increased risk and testing reductions did not lead to a large rise in new infections of the magnitude noted early in the HIV epidemic or a prevalence of infection that would be unmanageable for the South African health system (ie, a substantially higher prevalence than is currently managed). Incidence continued to fall, albeit at a slower rate than in the status quo scenario, and this slowing of the decline in infections could lead to appreciable further HIV infections. In the most extreme scenario that we modelled, cessation of general HIV testing services in 2025 and reducing condom use from 33% to 14% by 2035 resulted in 11 million more adult infections up to 2075—roughly equivalent to the number of people who acquired HIV in the past 40 years. Our analyses varying ART coverage and condom use in 2035 within 20 percentage points of their 2022 values model what we consider to be extreme but plausible changes in a time focused on maintaining coverage of effective interventions. ART coverage (among people aged 15–49 years) increased from 49% to 70% between 2015 and 2020, and condom use in South Africa is estimated to have increased from 4% to 26% between 1994 and 2004.^7^, ^9^, ^18^ All the scenarios we modelled are based on well managed changes to HIV testing programmes, in contrast with previous modelled scenarios created to warn against programme disruptions during COVID-19, in which rapid withdrawal of ART from people with HIV resulted in large immediate increases in infections and AIDS-related deaths.^19^

Some changes in programmatic outcomes that lead to changes in the overall population risk of HIV, such as changes in ART retention (affecting ART coverage) or uptake of preventive services, are routinely captured. Early detection of decreasing ART coverage could act as a trigger for reimplementation of general population testing or other interventions to mitigate accumulation of infection risk. Equally, monitoring of improvements in ART programme outcomes or declines in HIV incidence could enable more rapid scaling back of general population HIV testing or other intensive interventions without incurring adverse effects. However, other changes in underlying sexual risk (eg, changes in condom use, number of sexual partners, types of sexual contacts, or awareness of HIV within sexual partnerships) are more difficult to detect and might only be recognised via unexpected changes in the trajectory of the HIV epidemic over 5–10 years or more. This difficulty should encourage conservatism in decision making about HIV programme management. Changes in other outcomes, such as the incidence of some sexually transmitted infections (eg, gonorrhoea, chlamydia, acute syphilis), might become apparent more rapidly in response to changing sexual risk behaviours and could be useful proxy indicators for changes in the HIV risk environment^20^, ^21^ that could enable early HIV programme responses.

Our study considered the HIV epidemic in South Africa on a longer-term basis than most previous analyses, which projected that HIV incidence was unlikely to decline below one case per 1000 population per year up to 2035,^22^ or 2063.^23^ Another model suggested that universal test-and-treat (ie, annual testing of the entire population with immediate initiation of ART in people who test positive for HIV) could theoretically eliminate HIV in South Africa.^24^ The Thembisa model is well suited to long-term HIV epidemic projections because it incorporates detailed demography, is statistically calibrated to an extensive range of data sources, and incorporates heterogeneity in sexual behaviour, including in partner numbers, engagement in sex work, and sexual mixing. As the incidence of new HIV infections declines towards elimination, such heterogeneities and key population dynamics become increasingly important in mapping epidemic patterns.^8^, ^25^, ^26^ We did not consider the optimisation of HIV testing among specific modalities or population groups^6^, ^27^ or the potential effect of scaling up new preventive strategies (eg, long-acting injectable antiretrovirals, PrEP^28^) or other interventions optimised in the South Africa HIV Investment Case, which identifies the optimal portfolio of interventions against HIV within currently committed budget envelopes.^29^ Broadly, effective implementation of these strategies to accelerate declines in HIV incidence would affect our conclusions, similarly to the projected effects of increased condom use or ART coverage. Our projections are also somewhat more optimistic than modelling for Eswatini reported in 2020,^30^ which suggested that incidence would initially decline rapidly as ART coverage reached 90-90-90 targets, followed by steady but slower declines to 2050.

Our analysis had several additional limitations. Long-term projections require assumptions about future uncertainties that magnify as the time horizon increases. Our probabilistic analysis represented uncertainties in the parameters determining disease transmission, sexual behaviour, and treatment, and we considered the epidemiological effects of potential future changes in HIV programmes and sexual risk via scenario analysis. However, our modelling did not consider the full range of factors that could affect the epidemic in the long term, such as future technological advancements (eg, HIV vaccines or functional cures), changes in uptake of other preventive approaches (eg, voluntary medical male circumcision, PrEP), unexpected events that could affect the future of HIV programmes,^19^ or uncertainty about model structure.^31^ Projections of the epidemic to 2100 are therefore more uncertain than the calculated 95% CIs might suggest, and should be regarded as a projection of trends for investigative purposes, rather than a prediction of the future. Long-term programme-management decisions are largely driven by considerations of overall cost and efficiency relative to competing public health concerns. Our cost analysis showed how changes in HIV programmes and epidemiological outcomes translated to short-term and long-term HIV programme costs, but we did not quantify cost-effectiveness compared with other health-investment opportunities.

In conclusion, our modelling suggests that HIV programmes could probably scale back general population HIV testing without risking an immediate large increase in new infections or AIDS-related deaths, but that reductions in testing would result in only modest short-term savings and probably increase long-term costs. Therefore, HIV programmes face policy decisions about the opportunity to save or reallocate short-term resources by reducing testing, which needs to be balanced with potential slowing of reductions in the number of new HIV infections. Reduced testing combined with changes to the sexual risk environment could derail progress towards controlling HIV if programmatic changes happen too early, highlighting the need for caution in making large programmatic changes and continued monitoring to adjust programmes.

## Data sharing

No primary data were collected or reported in this study. Description of, and outputs from, Thembisa 4.5, and the code to reproduce our analyses are available online.

## Declaration of interests

LKW reports personal fees from Pacific Life Re and WHO, honoraria from *The Lancet Infectious Diseases* and the Luxembourg National Research Fund, and participation on a data safety monitoring board for the Wellcome Trust. LFJ reports participation on the scientific advisory board for the GIFT device (under development at the University of Cape Town, Cape Town, South Africa). JWI-E reports personal fees from BAO Systems and travel support from UNAIDS, the South African Centre for Epidemiological Modelling and Analysis, and the International AIDS Society. All other authors declare no competing interests.

## References

[1] UNAIDS (2015). Understanding fast-track: accelerating action to end the AIDS epidemic by 2030. https://www.unaids.org/en/resources/documents/2015/201506_JC2743_Understanding_FastTrack.

[2] UNAIDS (2023). The path that ends AIDS: UNAIDS global AIDS update 2023. https://www.unaids.org/en/resources/documents/2023/global-aids-update-2023.

[3] US President's Emergency Plan for AIDS Relief (2022).

[4] Stover J, Bollinger L, Izazola JA, Loures L, Delay P, Ghys PD (2016). What is required to end the AIDS epidemic as a public health threat by 2030? The cost and impact of the fast-track approach. PLoS One.

[5] UNAIDS (2022). UNAIDS data 2022. https://www.unaids.org/en/resources/documents/2023/2022_unaids_data.

[6] Johnson LF, Van Rensburg C, Govathson C, Meyer-Rath G (2019). Optimal HIV testing strategies for South Africa: a model-based evaluation of population-level impact and cost-effectiveness. Sci Rep.

[7] Johnson LF, Hallett TB, Rehle TM, Dorrington RE (2012). The effect of changes in condom usage and antiretroviral treatment coverage on human immunodeficiency virus incidence in South Africa: a model-based analysis. J R Soc Interface.

[8] Johnson LF, May MT, Dorrington RE (2017). Estimating the impact of antiretroviral treatment on adult mortality trends in South Africa: a mathematical modelling study. PLoS Med.

[9] Granich R, Gupta S, Hersh B (2015). Trends in AIDS deaths, new infections and ART coverage in the top 30 countries with the highest AIDS mortality burden, 1990–2013. PLoS One.

[10] Johnson LF, Dorrington RE (2022). Thembisa version 4.5: a model for evaluating the impact of HIV/AIDS in South Africa. https://thembisa.org/content/downloadPage/Thembisa4_5report.

[11] Granich RM, Gilks CF, Dye C, De Cock KM, Williams BG (2009). Universal voluntary HIV testing with immediate antiretroviral therapy as a strategy for elimination of HIV transmission: a mathematical model. Lancet.

[12] Meyer-Rath G, Brennan AT, Fox MP (2013). Rates and cost of hospitalisation before and after initiation of antiretroviral therapy in urban and rural settings in South Africa. J Acquir Immune Defic Syndr.

[13] US President's Emergency Plan for AIDS Relief (2022). PEPFAR 2022 country operational plan guidance. https://www.state.gov/2022-country-operational-plan-guidance/.

[14] Chamie G, Hickey MD, Kwarisiima D, Ayieko J, Kamya MR, Havlir DV (2020). Universal HIV testing and treatment (UTT) integrated with chronic disease screening and treatment: the SEARCH study. Curr HIV/AIDS Rep.

[15] Bekker L-G, Alleyne G, Baral S (2018). Advancing global health and strengthening the HIV response in the era of the Sustainable Development Goals: the International AIDS Society—*Lancet* Commission. Lancet.

[16] Grimsrud A, Wilkinson L, Ehrenkranz P (2023). The future of HIV testing in eastern and southern Africa: broader scope, targeted services. PLoS Med.

[17] Ong JJ, Coulthard K, Quinn C (2022). Risk-based screening tools to optimise HIV testing services: a systematic review. Curr HIV/AIDS Rep.

[18] Osler M, Hilderbrand K, Goemaere E (2018). The continuing burden of advanced HIV disease over 10 years of increasing antiretroviral therapy coverage in South Africa. Clin Infect Dis.

[19] Jewell BL, Smith JA, Hallett TB (2020). Understanding the impact of interruptions to HIV services during the COVID-19 pandemic: a modelling study. EClinicalMedicine.

[20] Hoornenborg E, Coyer L, Achterbergh RCA (2019). Sexual behaviour and incidence of HIV and sexually transmitted infections among men who have sex with men using daily and event-driven pre-exposure prophylaxis in AMPrEP: 2 year results from a demonstration study. Lancet HIV.

[21] Ayerdi Aguirrebengoa O, Vera García M, Arias Ramírez D (2021). Low use of condom and high STI incidence among men who have sex with men in PrEP programs. PLoS One.

[22] Johnson LF, Chiu C, Myer L (2016). Prospects for HIV control in South Africa: a model-based analysis. Glob Health Action.

[23] Abuelezam NN, McCormick AW, Fussell T (2016). Can the heterosexual HIV epidemic be eliminated in South Africa using combination prevention? A modeling analysis. Am J Epidemiol.

[24] Hontelez JAC, Lurie MN, Bärnighausen T (2013). Elimination of HIV in South Africa through expanded access to antiretroviral therapy: a model comparison study. PLoS Med.

[25] Stone J, Mukandavire C, Boily MC (2021). Estimating the contribution of key populations towards HIV transmission in South Africa. J Int AIDS Soc.

[26] Garnett GP. Reductions in HIV incidence are likely to increase the importance of key population programmes for HIV control in sub-Saharan Africa. *J Int AIDS Soc*; **24** (suppl 3): e25727. 10.1002/jia2.25727 PMC8242973 34189844

[27] Jamieson L, Johnson LF, Matsimela K (2021). The cost effectiveness and optimal configuration of HIV self-test distribution in South Africa: a model analysis. BMJ Glob Health.

[28] Jamieson L, Johnson LF, Nichols BE (2022). Relative cost-effectiveness of long-acting injectable cabotegravir versus oral pre-exposure prophylaxis in South Africa based on the HPTN 083 and HPTN 084 trials: a modelled economic evaluation and threshold analysis. Lancet HIV.

[29] Meyer-Rath G, Jamieson L, Johnson L (2021). South African HIV investment case—full report. https://www.heroza.org/wp-content/uploads/2021/12/HIV-Investment-Case-2021-Full-report-final.pdf.

[30] Akullian A, Morrison M, Garnett GP (2020). The effect of 90-90-90 on HIV-1 incidence and mortality in Eswatini: a mathematical modelling study. Lancet HIV.

[31] Eaton JW, Johnson LF, Salomon JA (2012). HIV treatment as prevention: systematic comparison of mathematical models of the potential impact of antiretroviral therapy on HIV incidence in South Africa. PLoS Med.

