# Supplementary material for: Future HIV epidemic trajectories in South Africa and projected long-term consequences of reductions in general population HIV testing: a mathematical modelling study
Source: Lancet Public Health. 2024 Mar 27;9(4):e218–30. doi: 10.1016/S2468-2667(24)00020-3 (PMC11000585; doi:10.1016/S2468-2667(24)00020-3)
Supplement: Supplementary appendix [file mmc1.pdf]

THE LANCET
Public Health

Supplementary appendix

This appendix formed part of the original submission and has been peer reviewed. We post it as supplied by the authors.

Supplement to: Rautenbach SP, Whittles LK, Meyer-Rath G, et al. Future HIV epidemic trajectories in South Africa and projected long-term consequences of reductions in general population HIV testing: a mathematical modelling study. *Lancet Public Health* 2024; **9:** e218–30.

Future HIV epidemic trajectories in South Africa and long-term consequences of reductions in general HIV testing: a mathematical modelling study — Supplementary Material

Stefan P. Rautenbach, Lilith K. Whittles, Gesine Meyer-Rath, Lise Jamieson, Thato Chidarikire, Leigh F. Johnson, Jeffrey W. Imai-Eaton

Correspondence to: stefan.rautenbach21@imperial.ac.uk; jeaton@hsph.harvard.edu

Table of contents

S1. Assumptions and calibration of testing and ART initiation in the Thembisa model 2
S2. Projection assumptions about male circumcision and pre-exposure prophylaxis 2
S3. Changes to Thembisa model to implement long-term projection scenarios 3
S4. Modelled scenarios 4
S5. Cost analysis assumptions 6
S6. Sensitivity of epidemic trajectory to timing of general HTS reductions 9
S7. Representative trajectories of future epidemiologic uncertainty 13
S8. Annual costs and distribution by component 19
S9. Additional indicators of epidemic trajectory under general HTS reductions 20
S10. Key population incidence, prevalence, and ART coverage under status quo 21
S11. References 22

S1. Assumptions and calibration of testing and ART initiation in the Thembisa model

Full description of the Thembisa model and its calibration used for this analysis are in the Thembisa 4.5 report: https://thembisa.org/content/downloadPage/Thembisa4_5report.[1]

The text below briefly describes key components of the model relevant to the current analysis, changes to the model implemented for this analysis, and refers to sections or tables and page numbers in the report where details can be found about specific components.

In the Thembisa model, individuals are exposed to monthly rates of HIV testing through different types of testing (Thembisa 4.5 report, Section 3.2, p. 29): (i) testing of pregnant women at antenatal clinics, (ii) testing prompted by opportunistic infections, (iii) testing of HIV-exposed infants, (iv) testing of partners of PLWH through passive partner notification, (v) self-testing, (vi) testing among PrEP clients (four times per year), and (vii) 'general' HTS (referred to as 'other HIV testing modalities' in the Thembisa 4.5 report). HIV testing rates vary by age, sex, previous HIV testing history, pregnancy rates, and opportunistic infection incidence. Testing rates vary by year to match reported numbers of HIV tests performed (Thembisa 4.5 report, Table B1, p. 151) and HIV prevalence among those tested (Thembisa 4.5 report, Table B3, p. 153). Rates of testing by age and sex are also calibrated to data from national household surveys on the percentage of adults reporting ever tested for HIV by sex, age group, and HIV status (Thembisa 4.5 report, Table B5, p. 154).[2] Modelled numbers of ANC testing are determined by numbers of births and ANC attendance. Opportunistic infection tests are based on modelled rates of opportunistic infection incidence and care seeking, which depend on CD4 infection stage of people with HIV (Thembisa 4.5 report, Table 3.1, p. 29). Passive partner notification testing (referred to as 'index HIV testing' in the Thembisa report) are determined by the number adults diagnosed through other modalities and proportion of newly diagnosed individuals whose partners are informed of their result and seek testing (Thembisa 4.5 report, Section B.5, p. 154). People retained on PrEP are assumed to test four times per year. To estimate the rate of general HTS among all adults, modelled numbers for adult HIV testing types (ANC, opportunistic infection care-seeking, passive partner notification, self-testing, PrEP, and other 'general HTS') are aggregated to calculate a predicted total number of adult tests and compared to data on total HIV tests conducted. In model calibration, rates of general HTS and are varied annually to derive rates consistent with total tests conducted and HIV prevalence. Thembisa 4.5 report, Sections B.6–B.10 (p. 157-165) contains technical details of model calibration and illustrates model fit to testing programme data and household survey data.

Following a positive HIV test, ART initiation rates depend on sex, CD4 count, and ART eligibility criteria, which change over time according to South Africa national guidelines (Thembisa 4.5 report, Table 3.3, p. 32). Initiation rates in each year are calibrated to match the total number of adults and children on ART programme scale-up. People on ART interrupt treatment at a time-constant rate that differs by sex but is independent of age, CD4 count, and ART duration. The female interruption rate was estimated during model calibration. The male rate was 1.2 times the female rate, based on previous studies.[3] The rate of resuming ART after interruption varied over time according to population HIV testing rates and by sex, with women resuming ART 1.5 times more rapidly than men.

Calibrated model parameters reflected the impact of the COVID-19 epidemic in South Africa. Rates were assumed to return to pre-COVID-19 levels by 2022 or 2023. General HIV testing rates from mid-2020 to mid-2021 were lower reflecting calibration to lower numbers of HIV tests conducted in 2020-21 (Thembisa 4.5 report, Table B1, p. 151). After 2022, testing rates returned to average testing rates over the 2016-2021 period. Rates of ART initiation following diagnosis were lower in 2020-21 in order to calibrate to lower numbers of new ART initiations (Thembisa 4.5 report, Table 3.4, p. 36). ART initiation rates returned to pre-2020 levels by 2023. Rates of voluntary medical male circumcision decreased in 2020-21 reflecting programme data reflecting lower numbers of circumcisions conducted (Thembisa 4.5 report, Table 4.5, p. 54). Higher non-HIV related mortality rates reflecting the direct impact of COVID-19 on mortality during 2019-20 and 2020-21 were derived from national excess mortality estimates (Thembisa 4.5 report, Section 6.3, p. 77).

S2. Projection assumptions about male circumcision and pre-exposure prophylaxis

Thembisa model projections incorporated continued provision of voluntary medical male circumcision (VMMC) and pre-exposure prophylaxis (PrEP) among higher risk populations. Male circumcision is assumed to lower male HIV acquisition by 60%, and has no effect on male-to-female or male-to-male HIV transmission rates. Circumcision rates represent both background traditional male circumcision, calibrated to circumcision data prior to 2008,[5] and VMMC implemented for HIV prevention. From 2021 onwards, the annual probability of VMMC in boys aged 10–14 years is assumed to be 0.26 per year and decreased with age to 0.15, 0.07, 0.036,

and 0.003 per year among age groups 15–19, 20–24, 25–49, and 50+ years, respectively. Under these assumptions, total circumcision coverage among men 15-49 years increased from 61% in 2021 to 91% in 2060 and was stable thereafter.

PrEP rollout was initiated in 2016 among FSW and among MSM in 2017. This was expanded to university students in 2018, adolescents and young women (AGYW) (15–24 years) from 2019, and the general population from 2021. In all population groups besides FSW, PrEP uptake is assumed to be proportional to the level of HIV risk, quantified by the product of the annual number of sexual partners (determined by age, risk group and marital status) and the HIV prevalence of those partners (determined by sex and key population status). From 2021–2025 the monthly PrEP initiation rate among PrEP eligible FSW is assumed to increase from 4.2% to 6.0% and remain at that rate until 2100. PrEP initiation rates among high-risk MSM aged 20 years and high-risk women aged 20 years who are PrEP eligible was assumed to be half that of FSW, increasing from 2.1% to 3.0% per month over 2021–2025. PrEP efficacy was assumed to be 65% in heterosexuals[6] and 85% in MSM.[7,8] Average duration of PrEP continuation was assumed to be 6-months among FSW and AGYW and one year among MSM. Under these PrEP initiation and continuation assumptions, the percentage of MSM on PrEP increased from 14% in 2021 to 42% in 2100 while the percentage of FSW on PrEP increased from 22% in 2021 to 34% in 2026 and was stable thereafter, and AGYW on PrEP increased from 5% in 2021 to 9% in 2026 and was stable thereafter.

S3. Changes to Thembisa model to implement long-term projection scenarios

Two changes were made to the Thembisa 4.5 model. Firstly, to extend Thembisa projections year to 2100, we changed the maximum projection years from 86 (1985 to 2070) to 116 (1985 to 2100) and extended relevant demographic, epidemiologic, and programme uptake parameters. Annual rates and proportions used in the input parameters were extended to 2100 assuming continuation of the same values that were constant between 2021 and 2070 up to 2100, including demographic rate parameters, time-varying HIV risk parameters (e.g. condom usage proportions), rates of HIV testing, care, and treatment, and uptake of male circumcision and PrEP for HIV prevention.

Demographic projection assumptions were retained as described in Section 6 of the Thembisa 4.5 model report (p. 72). Briefly, age-specific fertility and non-AIDS mortality rates were assumed to decline towards a set of asymptotic ultimate rates defined in the ASSA2008 model[9]. For fertility, the total fertility rate (TFR) declined from 2.4 in 2025 to the asymptotic level 1.7. For mortality, non-AIDS life expectancy increased towards an ultimate life expectancy of 82.1 years for men and 87.3 years for women. Mortality due to AIDS changed dynamically according to HIV incidence and treatment coverage. Net migration after 2022 tended towards zero at a rate of 4.5% per year. Under these assumptions the national population grew to a peak of 78 million in 2080, and slight decline to 77 million by 2100.

Secondly, we implemented changes to the model to scale the odds of condom use by relationship type by a time-varying odds ratio. In Thembisa 4.5, the probability of condom usage per sexual contact varies according to age, sex, relationship type, and knowledge of HIV positive status (Thembisa 4.5 report, Section 2.8, p. 17). Condom usage is highest in contacts between female sex workers (FSW) and their clients, then among short-term partnerships (ST; representing non-cohabiting/non-marital partnerships), and lowest among long-term partnerships (LT; representing cohabiting or marital partnerships). Rates of condom use over time are estimated from statistical analysis of nationally representative survey data (1986-2017) on reported condom use among females (Thembisa 4.5 report, Figure 2.3-2.4, p. 20) and studies reporting condom use among female sex workers (Thembisa 4.5 report, Table 2.5, p. 22), accounting for estimated reporting biases.[4]

In scenarios varying future levels of condom use over 2025–2035, the probability of condom use according to age/sex/relationship type/HIV knowledge was converted to the odds scale, the odds of condom use for all relationship types was scaled by the same amount for all contacts (linearly varying between 2025–2035), and then converted back to probability scale. The overall condom usage reported (‘total sex acts in which a condom is used’) represents the percentage of all sex acts in the population in which condoms were used, aggregated over all relationship types.

S4. Modelled scenarios

Tables S1–S4 summarise the scenarios that were performed for the analysis.

Table S1. Baseline

Testing rate	Testing reduction year	ART interruption rate	Condom usage odds
**2021 base rate**	N/A	2021 base rate	2021 base odds

Table S2. Testing reductions with status quo ART coverage and condom usage

Testing rate	Testing reduction year	ART interruption rate	Condom usage odds
**5–100% reduction**	2025	2021 base rate	2021 base odds
**5–100% reduction**	2030	2021 base rate	2021 base odds
**5–100% reduction**	2035	2021 base rate	2021 base odds
**5–100% reduction**	2040	2021 base rate	2021 base odds
**5–100% reduction**	2045	2021 base rate	2021 base odds
**5–100% reduction**	2050	2021 base rate	2021 base odds

Table S3. Testing reductions with altered ART coverage and condom usage

Testing rate	Testing reduction year	ART interruption rate	Condom usage odds
**2021 base rate**	N/A	0.5–14% increase over 10 years from 2025	2021 base odds
**2021 base rate**	N/A	0.5–14% decrease over 10 years from 2025	2021 base odds
**5–100% reduction**	2025	0.5–14% increase over 10 years from 2025	2021 base odds
**5–100% reduction**	2025	0.5–14% decrease over 10 years from 2025	2021 base odds
**5–100% reduction**	2030	0.5–14% increase over 10 years from 2025	2021 base odds
**5–100% reduction**	2030	0.5–14% decrease over 10 years from 2025	2021 base odds
**5–100% reduction**	2035	0.5–14% increase over 10 years from 2025	2021 base odds
**5–100% reduction**	2035	0.5–14% decrease over 10 years from 2025	2021 base odds
**5–100% reduction**	2040	0.5–14% increase over 10 years from 2025	2021 base odds
**5–100% reduction**	2040	0.5–14% decrease over 10 years from 2025	2021 base odds
**5–100% reduction**	2045	0.5–14% increase over 10 years from 2025	2021 base odds
**5–100% reduction**	2045	0.5–14% decrease over 10 years from 2025	2021 base odds
**5–100% reduction**	2050	0.5–14% increase over 10 years from 2025	2021 base odds
**5–100% reduction**	2050	0.5–14% decrease over 10 years from 2025	2021 base odds

Table S4. Testing reductions with status quo ART coverage and altered condom usage

Testing rate	Testing reduction year	ART interruption rate	Condom usage odds
**2021 base rate**	N/A	2021 base rate	0.5–14% increase over 10 years from 2025
**2021 base rate**	N/A	2021 base rate	0.5–14% decrease over 10 years from 2025
**5–100% reduction**	2025	2021 base rate	0.5–14% increase over 10 years from 2025
**5–100% reduction**	2025	2021 base rate	0.5–14% decrease over 10 years from 2025
**5–100% reduction**	2030	2021 base rate	0.5–14% increase over 10 years from 2025
**5–100% reduction**	2030	2021 base rate	0.5–14% decrease over 10 years from 2025
**5–100% reduction**	2035	2021 base rate	0.5–14% increase over 10 years from 2025
**5–100% reduction**	2035	2021 base rate	0.5–14% decrease over 10 years from 2025
**5–100% reduction**	2040	2021 base rate	0.5–14% increase over 10 years from 2025
**5–100% reduction**	2040	2021 base rate	0.5–14% decrease over 10 years from 2025
**5–100% reduction**	2045	2021 base rate	0.5–14% increase over 10 years from 2025
**5–100% reduction**	2045	2021 base rate	0.5–14% decrease over 10 years from 2025
**5–100% reduction**	2050	2021 base rate	0.5–14% increase over 10 years from 2025
**5–100% reduction**	2050	2021 base rate	0.5–14% decrease over 10 years from 2025

S5. Cost analysis assumptions

The cost analysis was performed by multiplying the units by the average cost per unit in Table S5. Assumptions on outputs used in the cost analysis are shown in Table S6. Costs were estimated from the providers' perspective (South African government) and presented in 2023 United States dollar (US$). Costs are calculated as fully-loaded fixed and variable costs, including the cost of staff time, consumables, lab tests (if applicable), drugs (if applicable), equipment, and overheads, as previously described in Meyer-Rath *et al. PLOS ONE* 2019.[10] Costs were inflated to 2023 prices for this analysis.

Table S5. Costs per unit for different intervention in SA HIV programme

Intervention	Programme Area	Average cost (2023 US$)[a]	Unit
**ART, Adults (1st line 1st year)**	Treatment	$272.03	per person per year
**ART, Adults (1st line follow-up)**	Treatment	$170.75	per person per year
**ART, Adults (2nd line follow-up)**	Treatment	$299.87	per person per year
**ART, Neonates**	Treatment	$91.24	per person per year
**ART, Children age <1 years old**	Treatment	$285.92	per person per year
**ART, Children 1–2 years (1st year)**	Treatment	$381.54	per person per year
**ART, Children 1–2 years (follow-up)**	Treatment	$311.45	per person per year
**ART, Children 3–5 years (1st year)**	Treatment	$441.98	per person per year
**ART, Children 3–5 years (follow-up)**	Treatment	$371.89	per person per year
**ART, Children 6–9 years (1st year)**	Treatment	$327.48	per person per year
**ART, Children 6–9 years (follow-up)**	Treatment	$257.38	per person per year
**ART, Children 10–14 years (1st year)**	Treatment	$263.07	per person per year
**ART, Children 10–14 years (follow-up)**	Treatment	$192.98	per person per year
**HIV testing, excluding ANC (negative)**	Testing	$3.62	per client tested
**HIV testing, excluding ANC (positive)**	Testing	$5.20	per client tested
**ANC testing (negative)**	Testing	$3.16	per client tested
**ANC testing (positive)**	Testing	$4.72	per client tested
**Palliative care (home-based)**	Inpatient	$65.42	per AIDS-death
**Inpatient care (ART; <200 cells/μl)**	Inpatient	$399.63	per patient year
**Inpatient care (ART; 200–349 cells/μl)**	Inpatient	$126.16	per patient year
**Inpatient care (ART; 350–500 cells/μl)**	Inpatient	$37.20	per patient year
**Inpatient care (ART; >500 cells/μl)**	Inpatient	$37.20	per patient year
**Inpatient care (pre-ART; <200 cells/μl)**	Inpatient	$155.56	per patient year
**Inpatient care (pre-ART; 200–349 cells/μl)**	Inpatient	$83.43	per patient year
**Inpatient care (pre-ART; 350–500 cells/μl)**	Inpatient	$46.57	per patient year
**Inpatient care (pre-ART; >500 cells/μl)**	Inpatient	$46.57	per patient year

[a] ART and inpatient care costs are from Meyer-Rath *et al. PLOS ONE* 2019.[10] HIV testing costs are from Johnson *et al. Scientific Reports* 2019.[11] Costs were inflated to 2023 prices for this analysis.

Table S6. Assumptions applied to cost analysis

Cost units	Assumption
**ART, Adults (1st line 1st year)**	The annual number of adults (15+ years) starting ART.
**ART, Adults (1st line follow-up)**	The annual number of adults (15+ years) on ART minus the sum of the annual number of adults (15+ years) starting ART and the annual number of adults (15+ years) on second line ART.
**ART (2nd line follow-up)**	The annual number of adults (15+ years) on second line ART per year.
**ART, Neonates**	The annual number of neonates (infants born with HIV) on ART.
**ART, Children age <1 years old**	The annual number of children (<1 years) on ART.
**ART, Children 1–2 years (1st year)**	The annual number of children (1–2 years) starting ART.
**ART, Children 1–2 years (follow-up)**	The annual number of children (1–2 years) on ART per year minus the annual number of children (1–2 years) starting ART.
**ART, Children 3–5 years (1st year)**	The annual number of children (3–5 years) starting ART.
**ART, Children 3–5 years (follow-up)**	The annual number of children (3–5 years) on ART minus the annual number of children (3–5 years) starting ART.
**ART, Children 6–9 years (1st year)**	The annual number of children (6–9 years) starting ART.
**ART, Children 6–9 years (follow-up)**	The annual number of children (6–9 years) on ART minus the annual number of children (6–9 years) starting ART.
**ART, Children 10–14 years (1st year)**	The number of children (10–14 years) starting ART.
**ART, Children 10–14 years (follow-up)**	The annual number of children (10–14 years) on ART minus the annual number of children (10–14 years) starting ART.
**HIV testing, excluding ANC (negative)**	The annual number of negative HIV tests amongst all ages performed outside of ANC (including general HTS, OI patient testing, passive partner notification, PrEP clients).
**HIV testing, excluding ANC (positive)**	The annual number of positive HIV tests amongst all ages performed outside ANC (including general HTS, OI patient testing, passive partner notification, PrEP clients).
**ANC Testing (negative)**	The annual number of positive tests amongst pregnant women at ANC.
**ANC Testing (positive)**	The annual number of negative tests amongst pregnant women at ANC.
**Palliative care (home-based)**	The annual number of AIDS-related deaths amongst all ages.
**Inpatient care (ART; <200 cells/µl)**	The annual number of adults (15+ years) with HIV on ART with CD4 count <200 cells/µl.
**Inpatient care (ART; 200–349 cells/µl)**	The annual number of adults (15+ years) with HIV on ART with CD4 count 200–349 cells/µl.
**Inpatient care (ART; 349–500 cells/µl)**	The annual number of adults (15+ years) with HIV on ART with CD4 count 349–500 cells/µl.
**Inpatient care (ART; >500 cells/µl)**	The annual number of adults (15+ years) with HIV on ART with CD4 count >500 cells/µl.
**Inpatient care (pre-ART; <200 cells/µl)**	The annual number of adults (15+ years) with HIV not on ART with CD4 count <200 cells/µl. There are no separate costs for those who discontinue ART. We assumed that those who discontinue ART with CD4 count <200 cells/µl would have the same inpatient costs as

	those who are pre-ART with CD4 count <200 cells/µl and added the numbers of those who discontinued to the pre-ART numbers.
**Inpatient care (pre-ART; 200–349 cells/µl)**	The annual number of adults (15+ years) with HIV not on ART with CD4 count 200–349 cells/µl. There are no separate costs for those who discontinue ART. We assumed that those who discontinue ART with CD4 count 200–349 cells/µl would have the same inpatient costs as those who are pre-ART with CD4 count 200–349 cells/µl and added the numbers of those who discontinued to the pre-ART numbers.
**Inpatient care (pre-ART; 349–500 cells/µl)**	The annual number of adults (15+ years) with HIV not on ART with CD4 count 349–500 cells/µl. There are no separate costs for those who discontinue ART. We assumed that those who discontinue ART with CD4 count 349–500 cells/µl would have the same inpatient costs as those who are pre-ART with CD4 count 349–500 cells/µl and added the numbers of those who discontinued to the pre-ART numbers.
**Inpatient care (pre-ART; >500 cells/µl)**	The annual number of adults (15+ years) with HIV not on ART with CD4 count >500 cells/µl. There are no separate costs for those who discontinue ART. We assumed that those who discontinue ART with CD4 count >500 cells/µl would have the same inpatient costs as those who are pre-ART with CD4 count >500 cells/µl and added the numbers of those who discontinued to the pre-ART numbers.

S6. Sensitivity of epidemic trajectory to timing of general HTS reductions

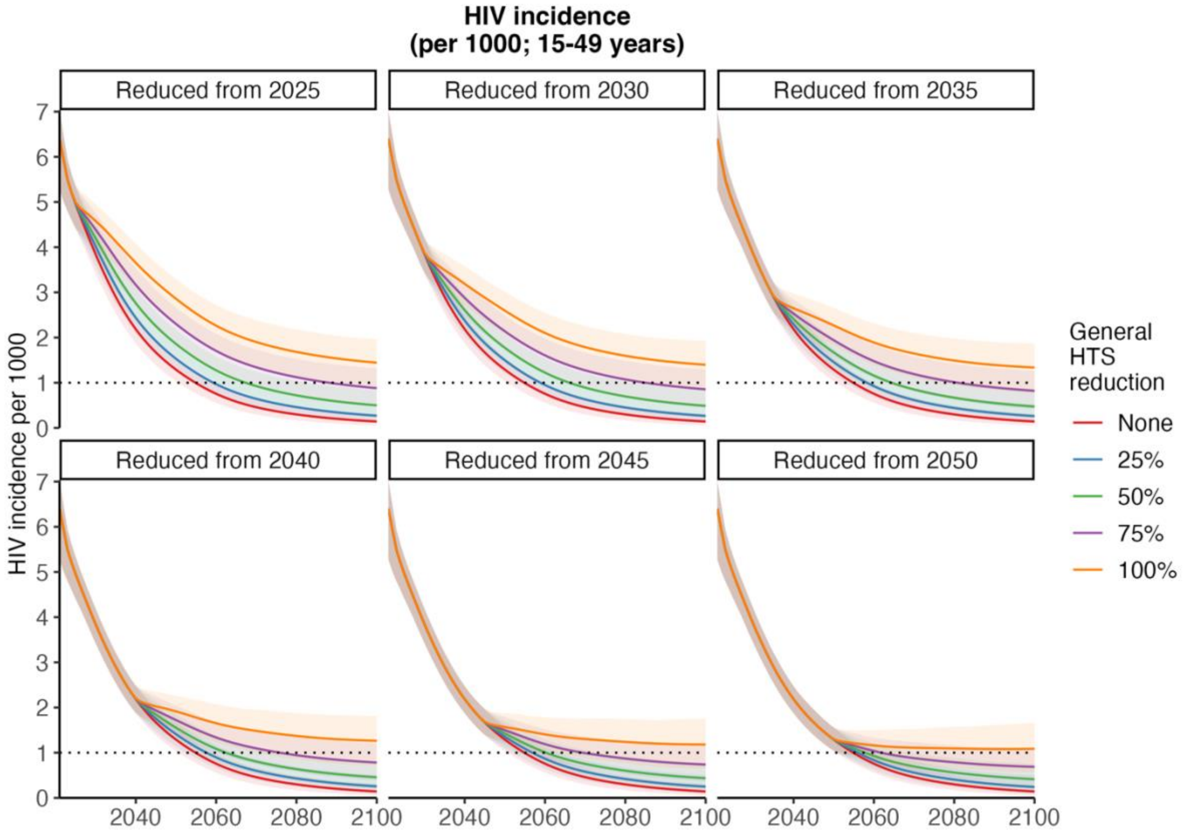

Figure S1: Sensitivity of HIV incidence to timing of general HTS reductions. Changes in incidence over time when general HTS was reduced from different time points. Figures of HIV incidence rate (15–49 years) per 1000 (mean and 95% CI) including an indication of when 'elimination' was attained (dotted line) between 2020 and 2100. Lines show status quo (no testing reduction) and general HTS reductions of 25%, 50%, 75% and 100% when testing was reduced from 2025, 2030, 2035, 2040, 2045 and 2050.

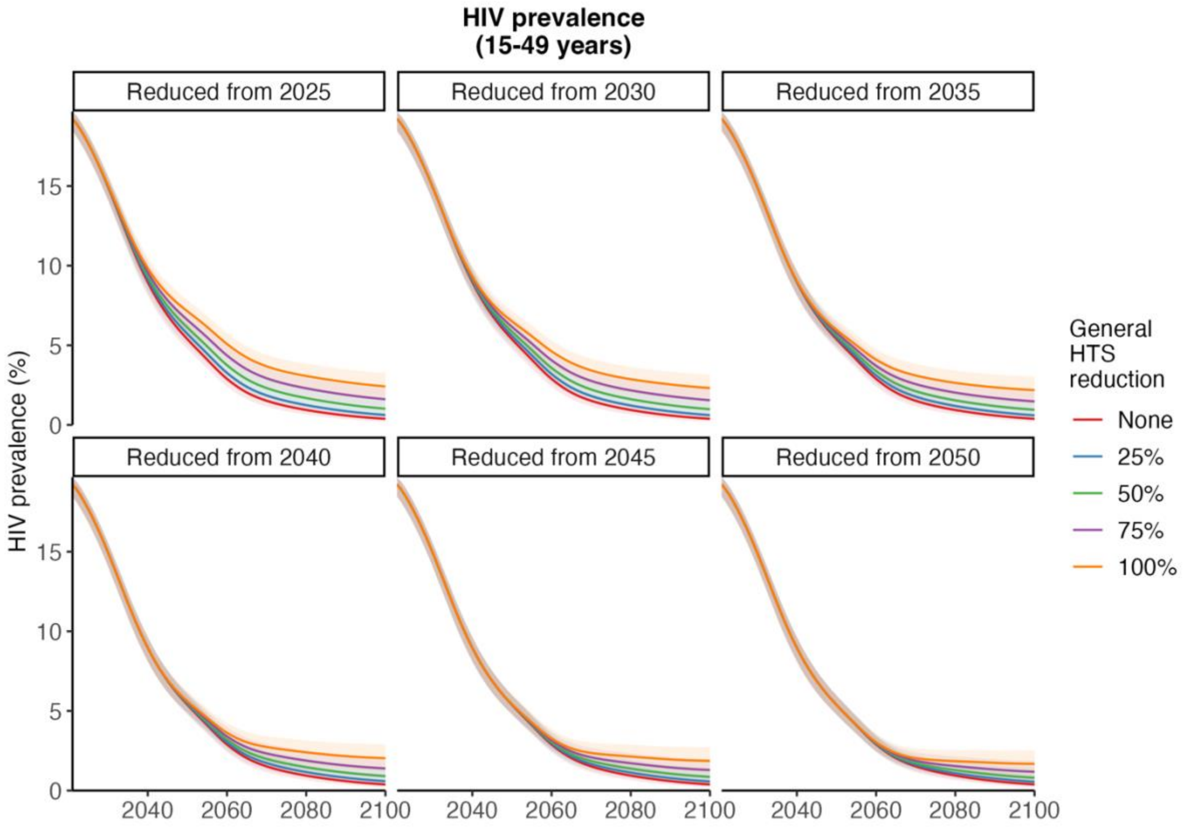

Figure S2: Sensitivity of HIV prevalence to timing of general HTS reductions. Changes in HIV prevalence when general HTS was reduced from different time points. Figures of HIV prevalence (%) (15–49 years) (mean and 95% CI) between 2020 and 2100. Lines show status quo (no testing reduction) and general HTS reductions of 25%, 50%, 75% and 100% when testing was reduced from 2025, 2030, 2035, 2040, 2045 and 2050.

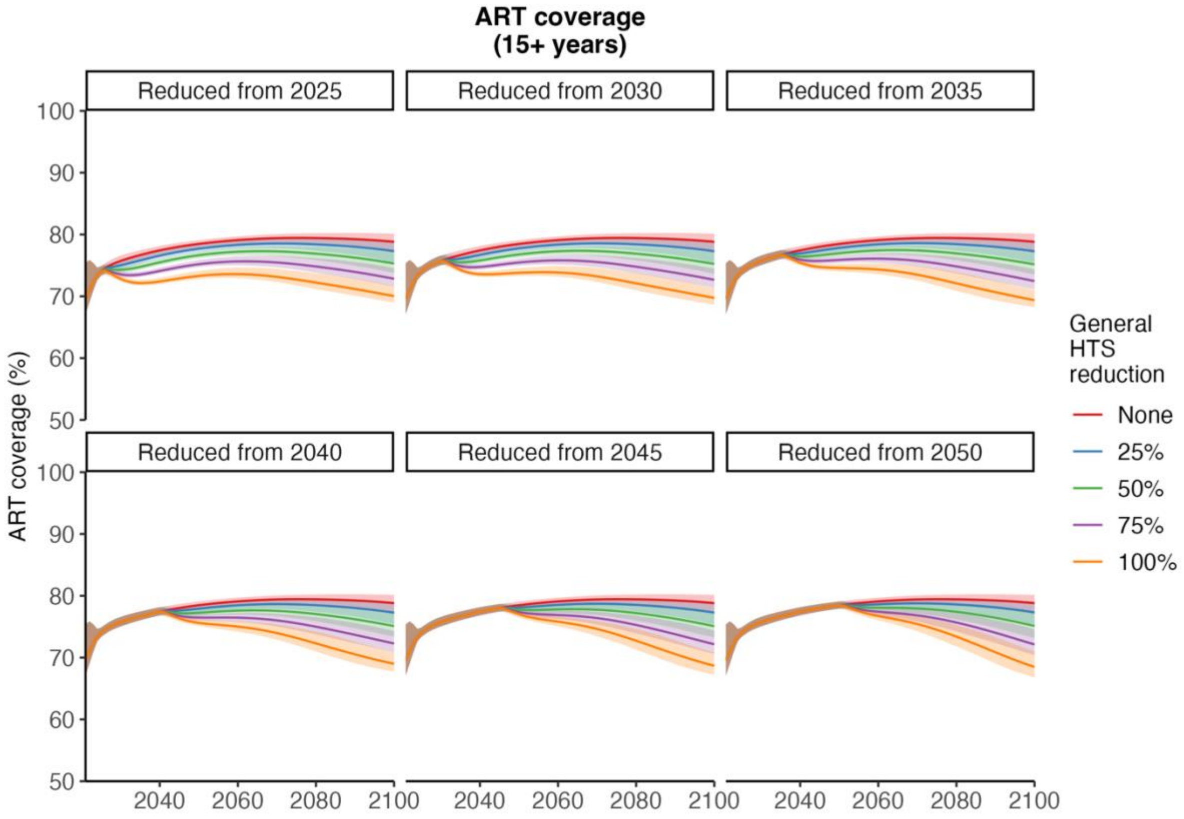

Figure S3: Sensitivity of ART coverage to timing of general HTS reductions. Changes in ART coverage when general HTS was reduced from different time points. Figures of ART coverage (%) (over 15 years) (mean and 95% CI) between 2020 and 2100. Lines show status quo (no testing reduction) and general HTS reductions of 25%, 50%, 75% and 100% when testing was reduced from 2025, 2030, 2035, 2040, 2045 and 2050.

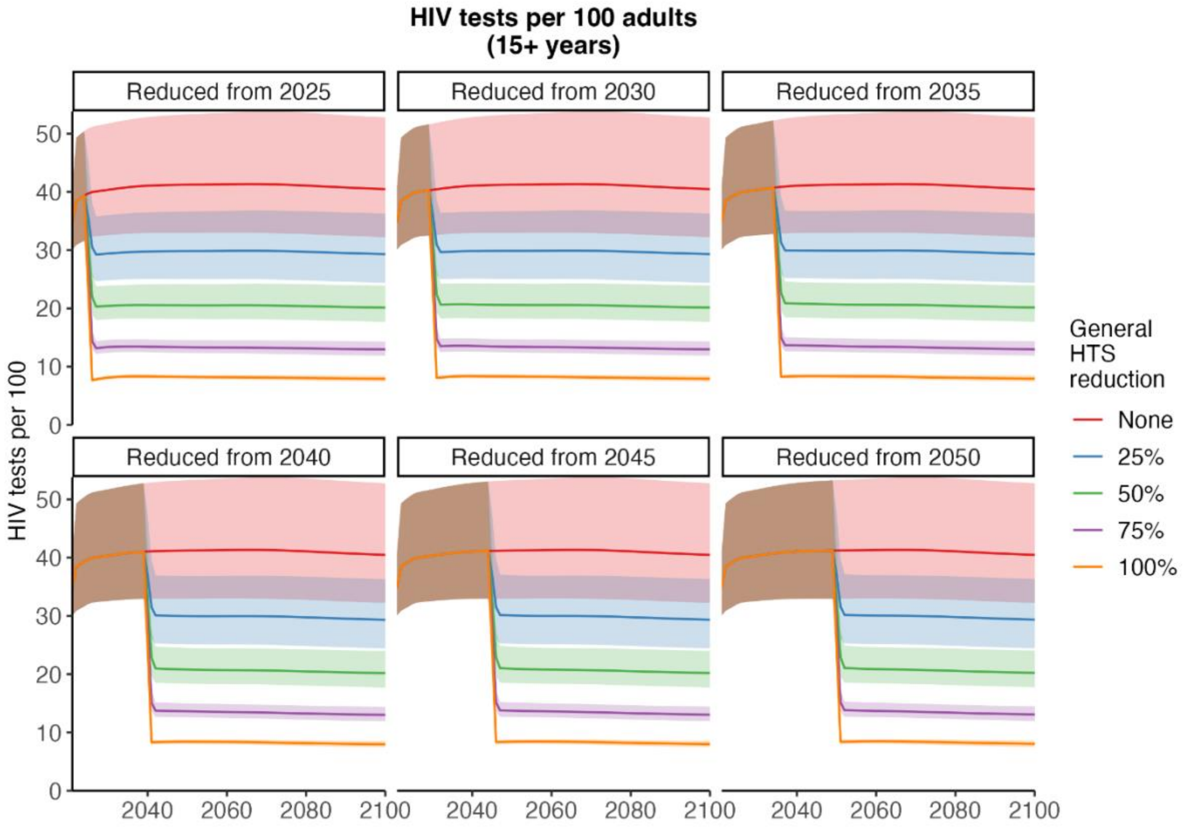

Figure S4: Sensitivity of HIV testing to timing of general HTS reductions. Changes in HIV tests when general HTS was reduced from different time points. Figures of HIV tests per 1000 adults (over 15 years) (mean and 95% CI) between 2020 and 2100. Lines show status quo (no testing reduction) and general HTS reductions of 25%, 50%, 75% and 100% when testing was reduced from 2025, 2030, 2035, 2040, 2045 and 2050.

S7. Representative trajectories of future epidemiologic uncertainty

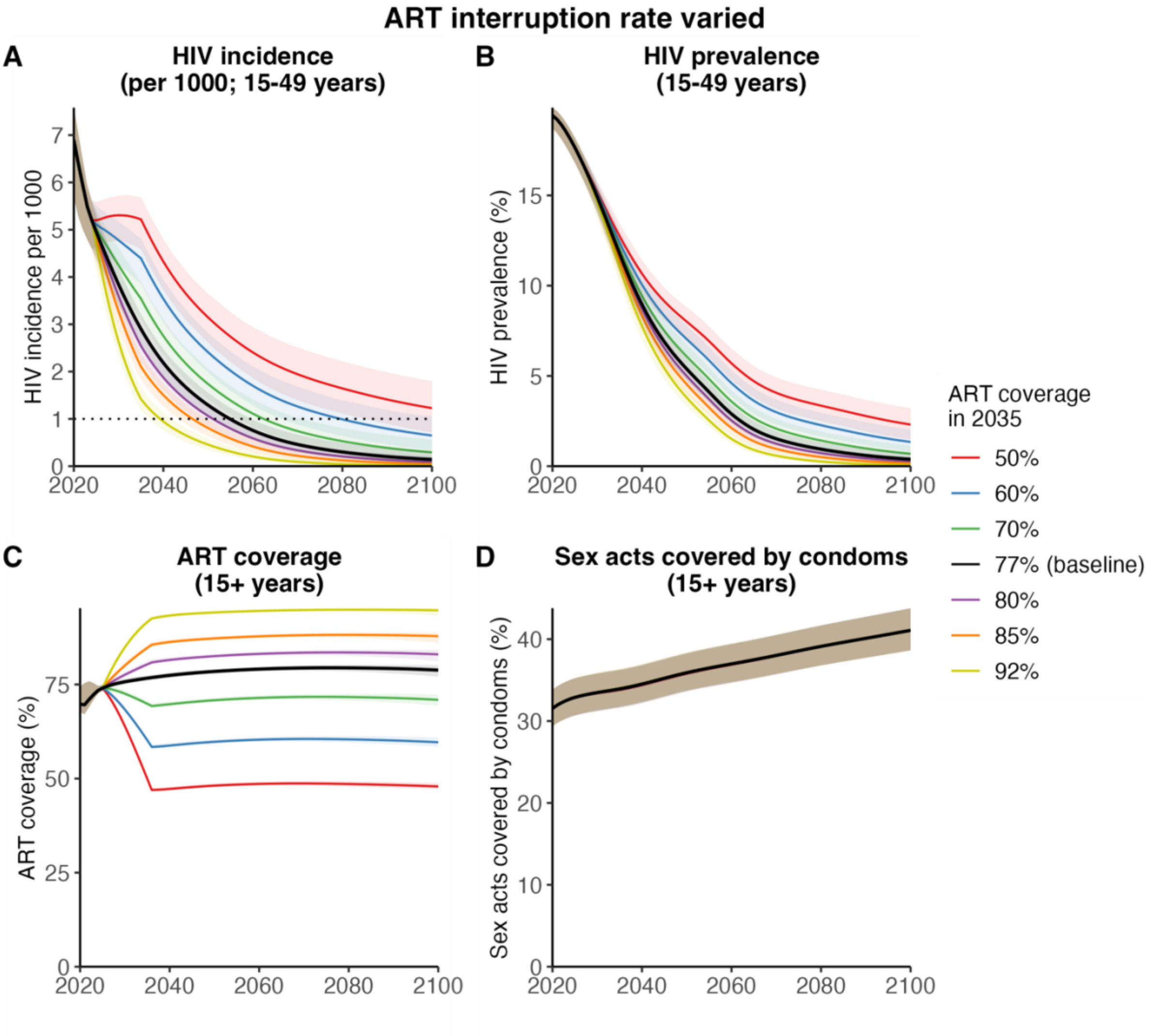

Figure S5: Sensitivity of HIV incidence and prevalence to future changes in ART interruption rate. Changes in incidence, prevalence, ART coverage and condom usage over time when ART interruption increased or decreased. Changes in the odds of ART interruption were implemented linearly over the period 2025–2035. Representative figures of HIV incidence rate (15–49 years) (A) per 1000 (mean and 95% CI) including an indication of when ‘virtual elimination’ (incidence <1/1000) was attained (dotted line), HIV prevalence (15–49 years) (B), ART coverage of adults (over 15 years) (C) and condom usage (%) (over 15 years) (D) between 2020 and 2100 showing status quo testing when ART coverage in 2035 was either retained at baseline (77%), increased to 80%, 85% or 92%, or decreased to 70%, 60%, or 50%.

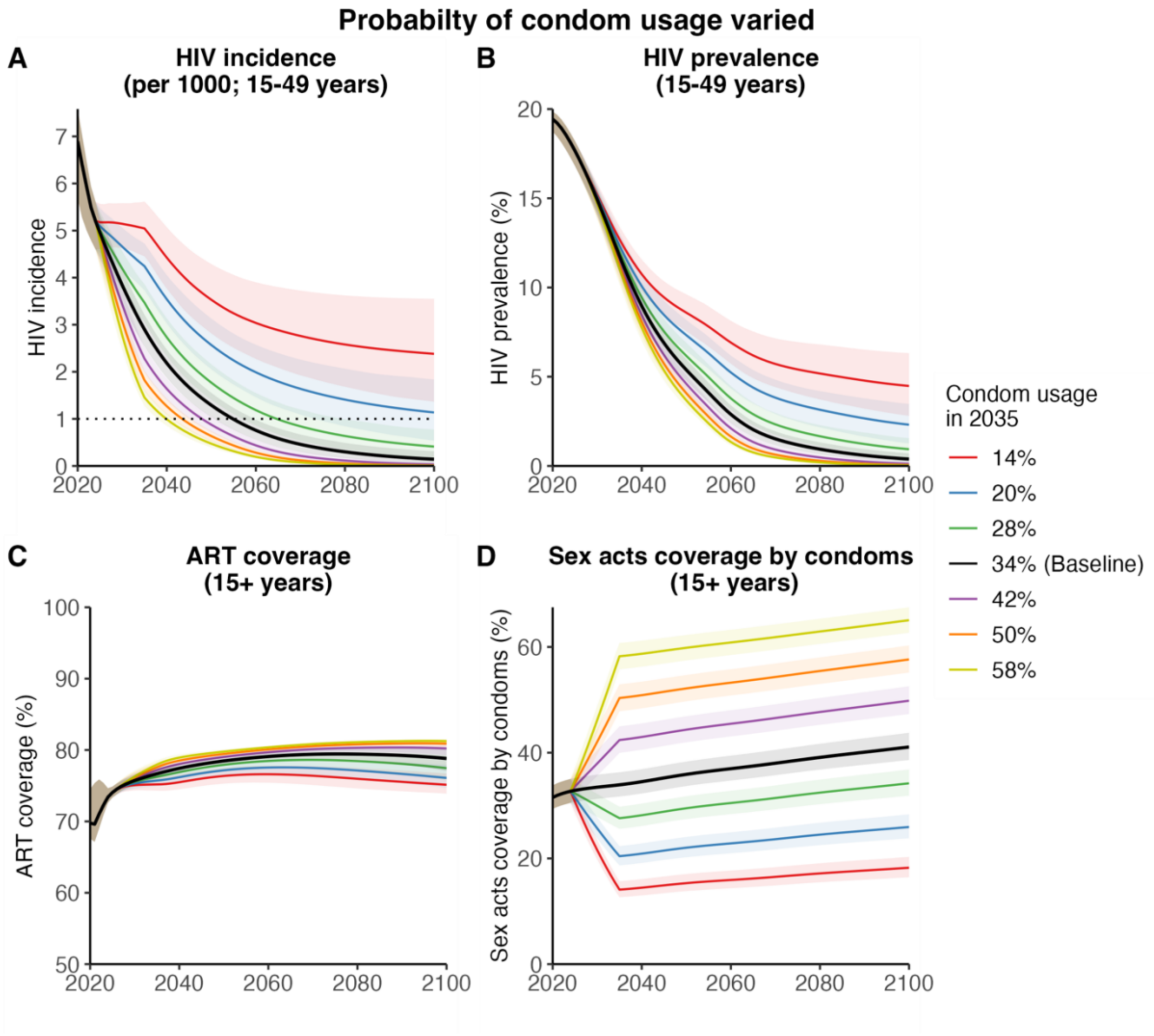

Figure S6: Sensitivity of HIV incidence and prevalence to future changes in condom usage. Changes in incidence, prevalence, ART coverage and condom usage over time when condom usage increased or decreased. Changes in the odds of condom usage were implemented linearly over the period 2025–2035.Representative figures of HIV incidence rate (15–49 years) (A) per 1000 (mean and 95% CI) including an indication of when 'virtual elimination' (incidence <1/1000) was attained (dotted line), HIV prevalence (15–49 years) (B), ART coverage of adults (over 15 years) (C) and condom usage (%) (over 15 years) (D) between 2020 and 2100 showing status quo testing when ART coverage in 2035 was either retained at baseline (77%), increased to 80%, 85% or 92%, or decreased to 70%, 60%, or 50%.

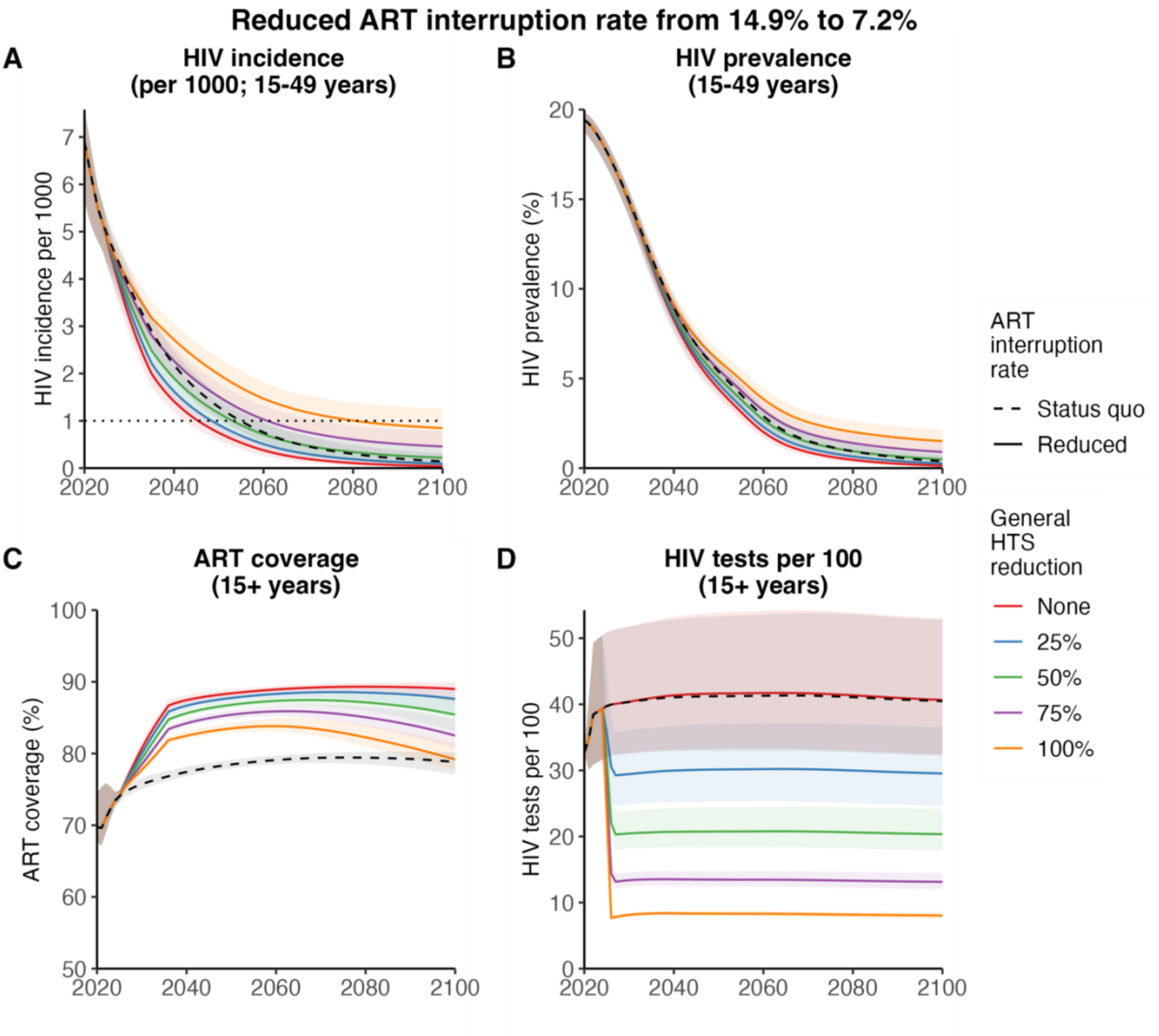

Figure S7: Representative scenario in which ART interruption rate reduced from 14.9% to 7.2% per year between 2025–2035. Changes in incidence, prevalence, ART coverage and HIV tests over time when general HTS was reduced, and ART interruption reduced. Changes in the odds of ART interruption were implemented linearly over the period 2025–2035. Representative figures of HIV incidence rate (15–49 years) (A) per 1000 (mean and 95% CI) including an indication of when 'virtual elimination' (incidence <1/1000) was attained (dotted line), HIV prevalence (15–49 years) (B), ART coverage of adults (over 15 years) (C) and HIV tests per 100 (over 15 years) (D) between 2020 and 2100 showing status quo testing and treatment (ART coverage in 2035 of 76.7%), no testing reduction (ART coverage in 2035 increased to 85.8%) and general HTS reductions of 25%, 50%, 75% and 100% from 2025 (ART coverage in 2035 increased to 85.8%).

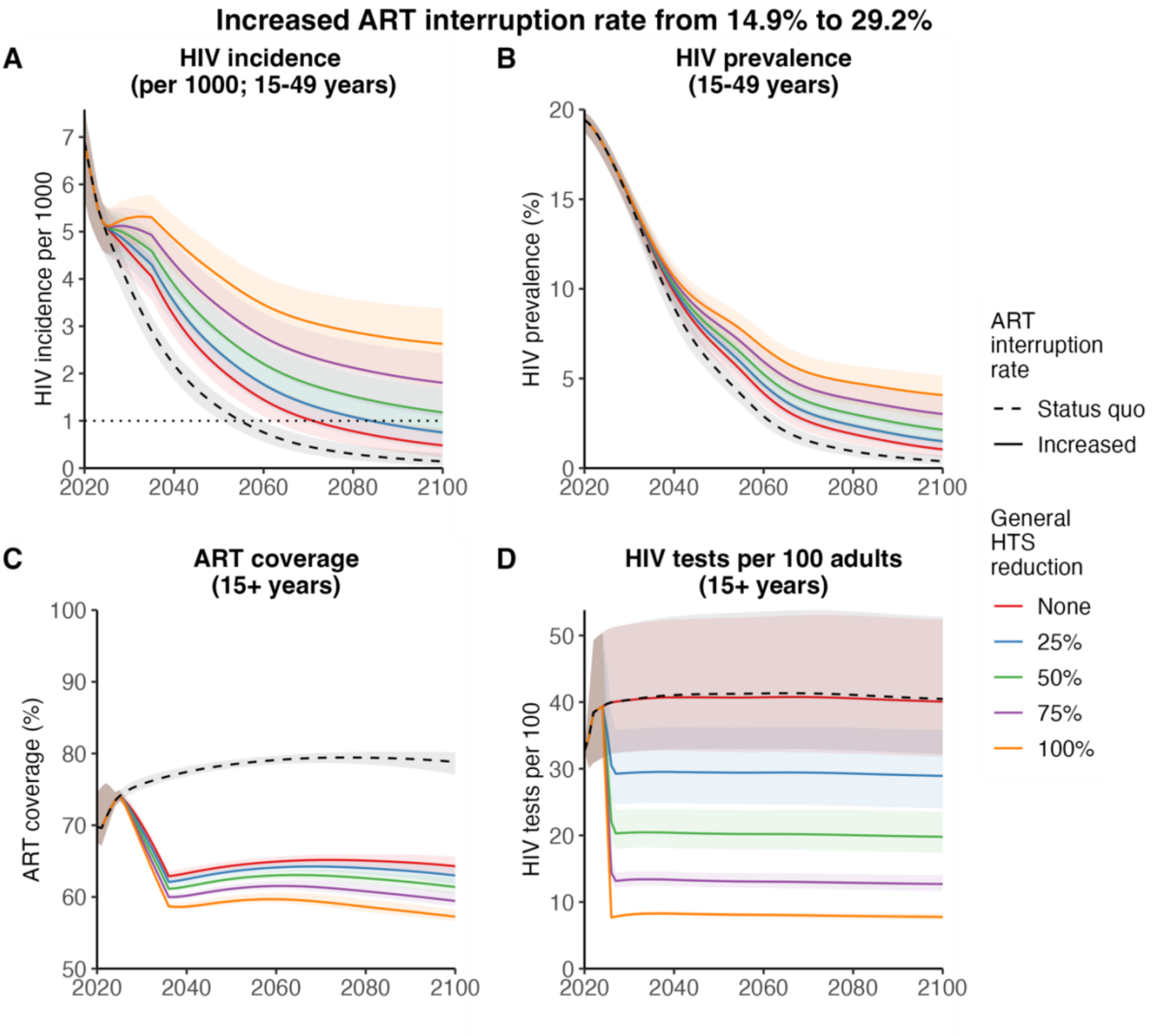

Figure S8: Representative scenario in which ART interruption rate increased from 14.9% to 29.2% per year between 2025–2035. Changes in incidence, prevalence, ART coverage and HIV tests over time when general HTS was reduced, and ART interruption increased. Changes in the odds of ART interruption were implemented linearly over the period 2025–2035. Representative figures of HIV incidence rate (15–49 years) (A) per 1000 (mean and 95% CI) including an indication of when 'virtual elimination' (incidence <1/1000) was attained (dotted line), HIV prevalence (15–49 years) (B), ART coverage of adults (over 15 years) (C) and HIV tests per 100 (over 15 years) (D) between 2020 and 2100 showing status quo testing and treatment (ART coverage in 2035 of 76.7%), no testing reduction (ART coverage in 2035 decreased to 64.2%) and general HTS reductions of 25%, 50%, 75% and 100% from 2025 (ART coverage in 2035 decreased to 64.2%).

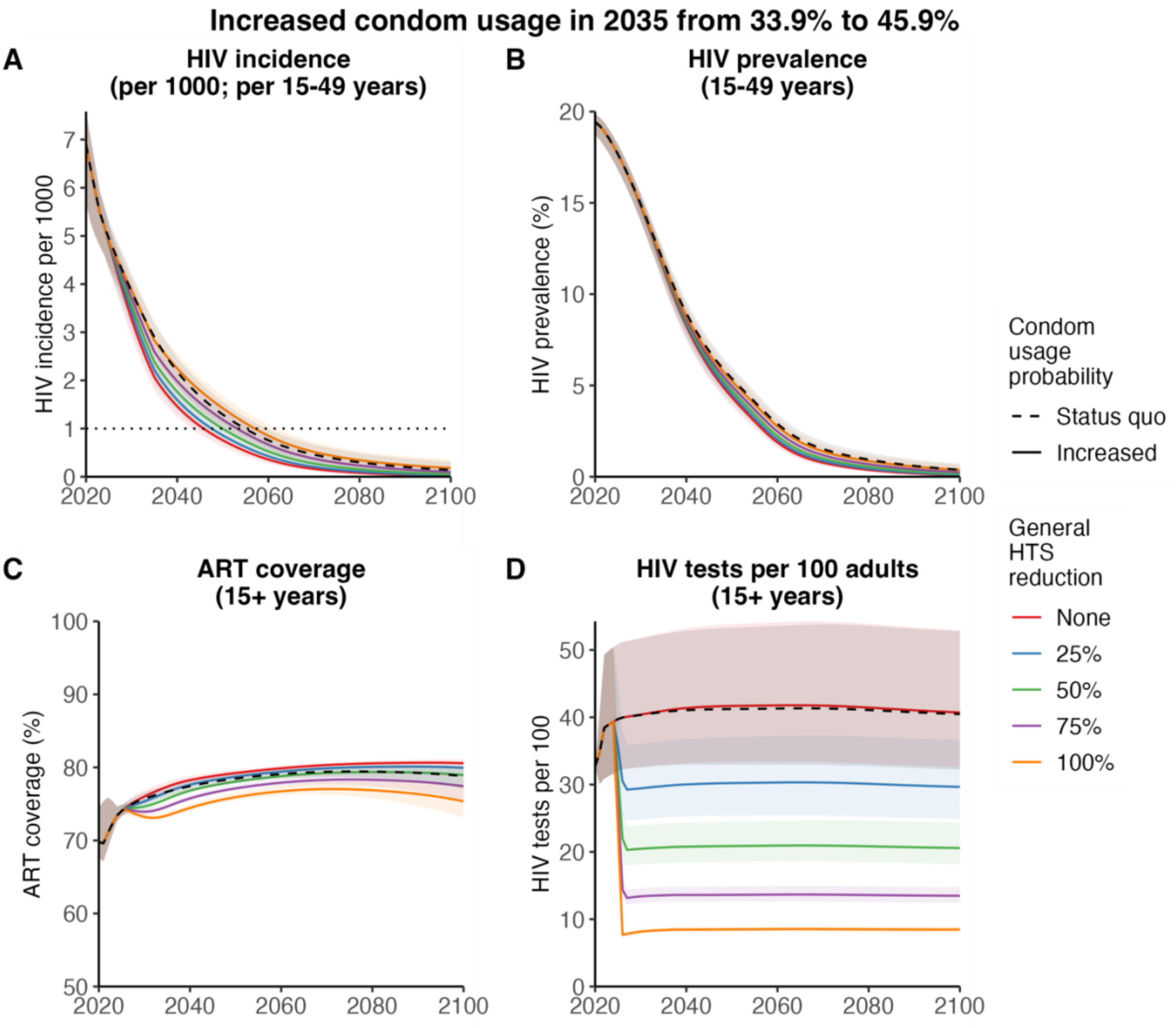

Figure S9: Representative scenario in which proportion of sex acts covered by condoms increased from 33.9% to 45.9% between 2025–2035. Changes in incidence, prevalence, ART coverage and HIV tests over time when general HTS was reduced, and condom usage increased. Changes in the odds of condom usage were implemented linearly over the period 2025–2035. Representative figures of HIV incidence rate (15–49 years) (A) per 1000 (mean and 95% CI) including an indication of when 'virtual elimination' (incidence <1/1000) was attained (dotted line), HIV prevalence (15–49 years) (B), ART coverage of adults (over 15 years) (C) and HIV tests per 100 (over 15 years) (D) between 2020 and 2100 showing status quo testing and condom usage (condom usage in 2035 of 33.9%), no testing reduction (condom usage in 2035 increased to 45.9%) and general HTS reductions of 25%, 50%, 75% and 100% from 2025 (condom usage in 2035 increased to 45.9%).

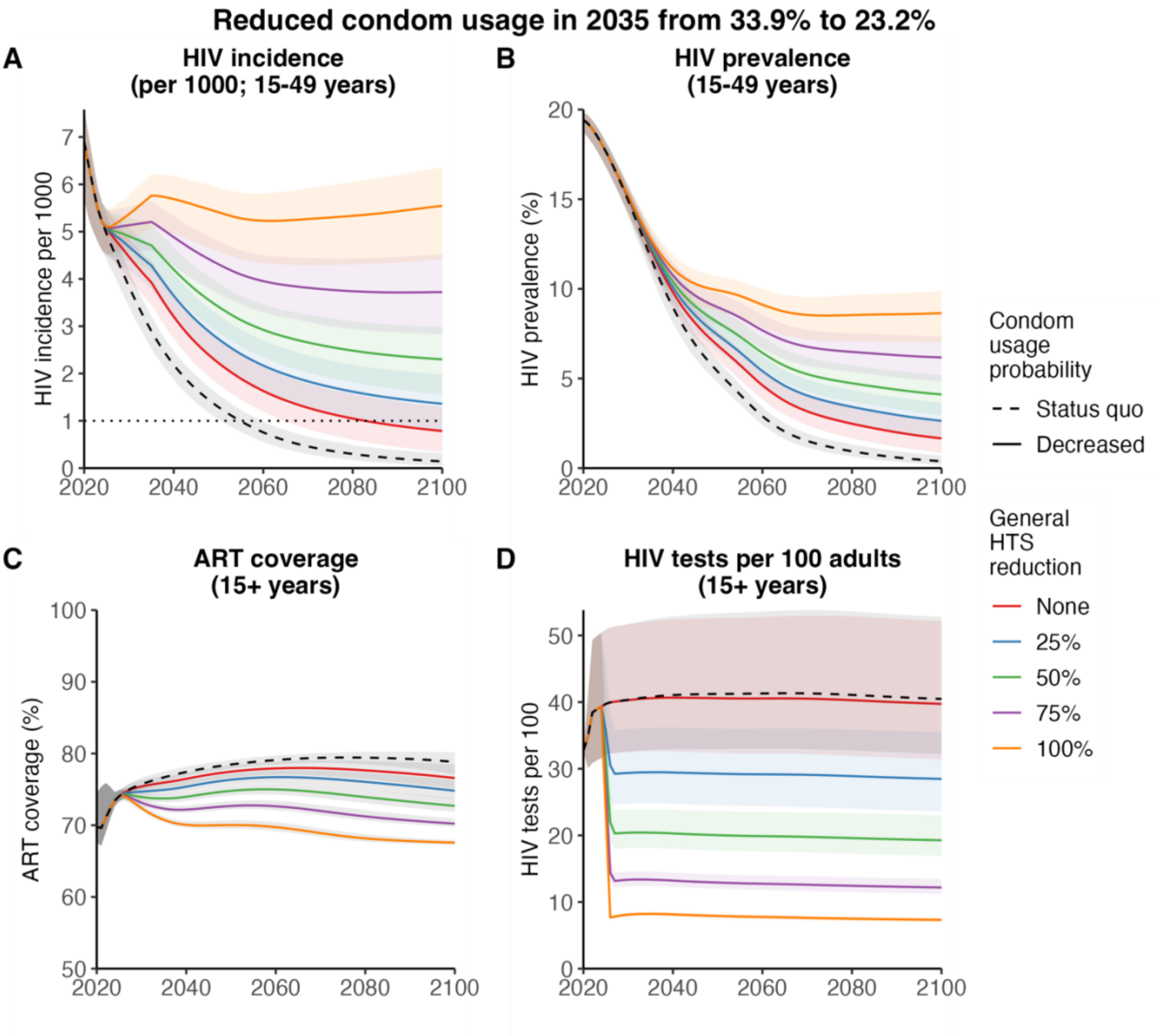

Figure S10: Representative scenario in which proportion of sex acts covered by condoms reduced from 33.9% to 23.2% between 2025–2035. Changes in incidence, prevalence, ART coverage and HIV tests over time when condom usage decreased. Changes in the odds of condom usage were implemented linearly over the period 2025–2035. Representative figures of HIV incidence rate (15–49 years) (A) per 1000 (mean and 95% CI) including an indication of when 'virtual elimination' (incidence <1/1000) was attained (dotted line), HIV prevalence (15–49 years) (B), ART coverage of adults (over 15 years) (C) and HIV tests per 100 (over 15 years) (D) between 2020 and 2100 showing status quo testing and condom usage (condom usage in 2035 of 33.9%), no testing reduction (condom usage in 2035 decreased to 23.2%) and general HTS reductions of 25%, 50%, 75% and 100% from 2025 (condom usage in 2035 decreased to 23.2%).

S8. Annual costs and distribution by component

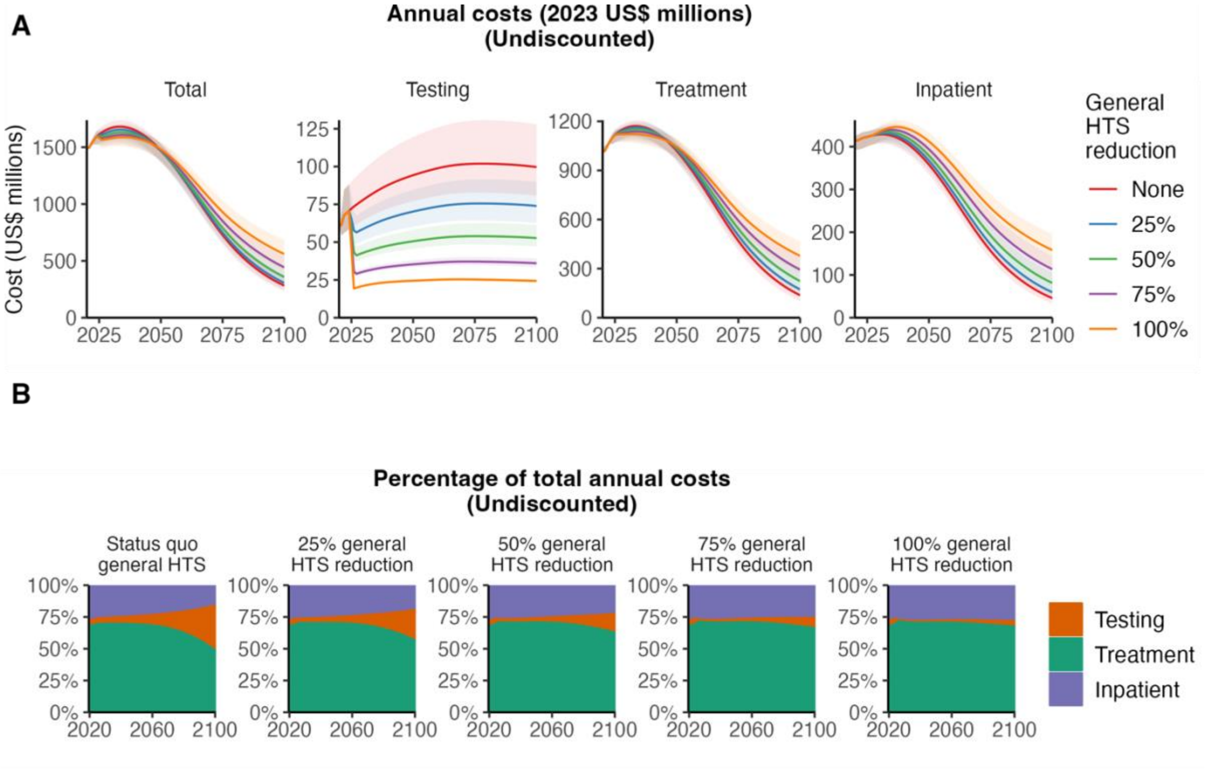

Figure S11: Annual HIV testing and treatment programme costs by category over 2025–2100. (A) Annual cost for total HIV testing and treatment programme and cost for testing, treatment, and care under status quo scenario and strategies reducing general HTS by 25%, 50% 75%, and 100%. (B) Distribution of total testing and treatment programme cost towards HIV testing, treatment, and care over time under each scenario.

S9. Additional indicators of epidemic trajectory under general HTS reductions

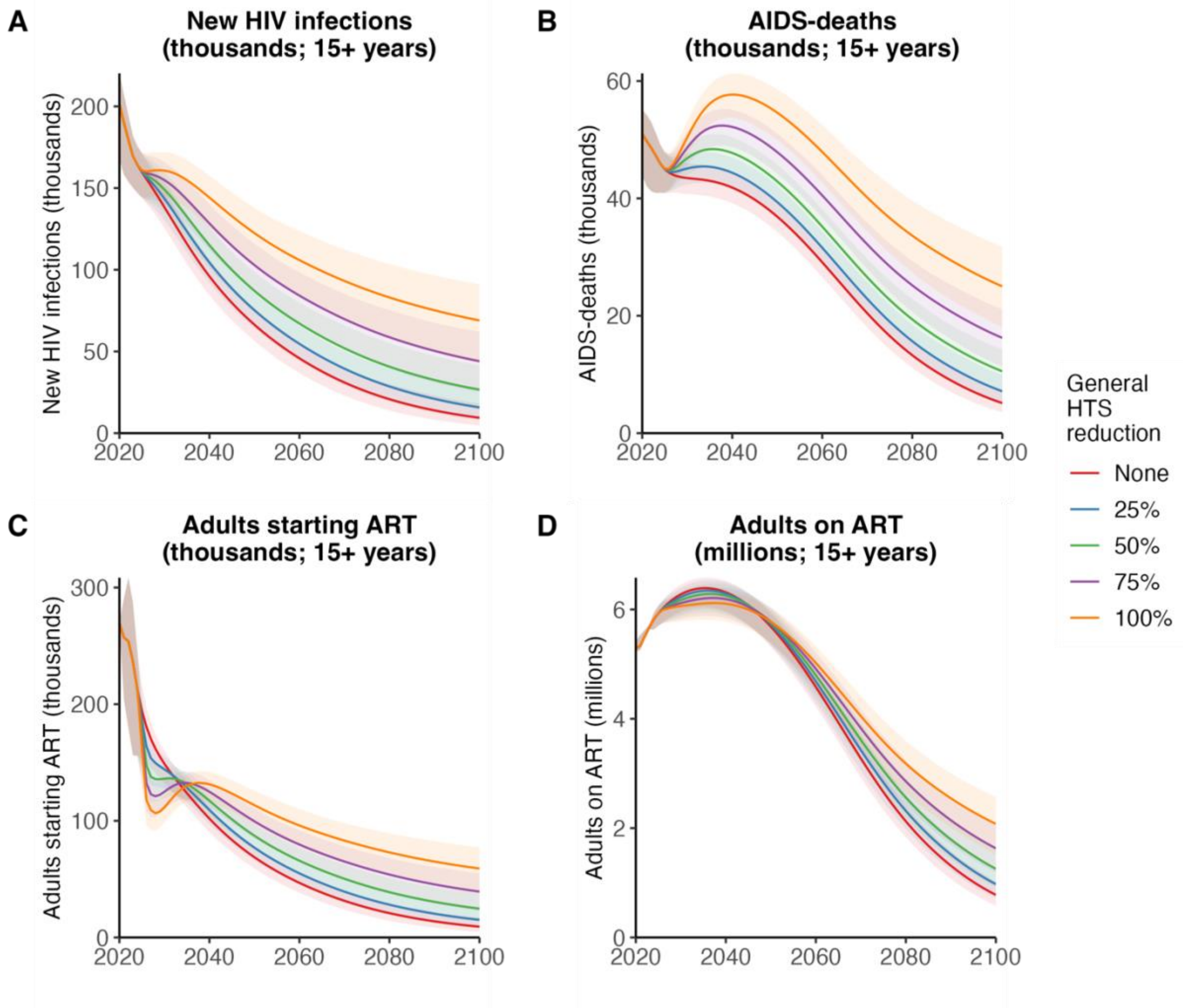

Figure S12. Changes in new HIV infections, AIDS-deaths, adults starting ART and adults on ART over time when general HTS was reduced. Numbers of (A) New HIV infections (over 15 years), (B) AIDS-related deaths (over 15 years), (C) adults starting ART (over 15 years), and (D) total adults on ART (over 15 years) between 2020 and 2100. Figures represent status quo scenario (no testing reduction) and general HTS reductions of 25%, 50%, 75% and 100% implemented in 2025. Lines represent posterior mean and shaded areas are 95% credible intervals.

S10. Key population incidence, prevalence, and ART coverage under status quo

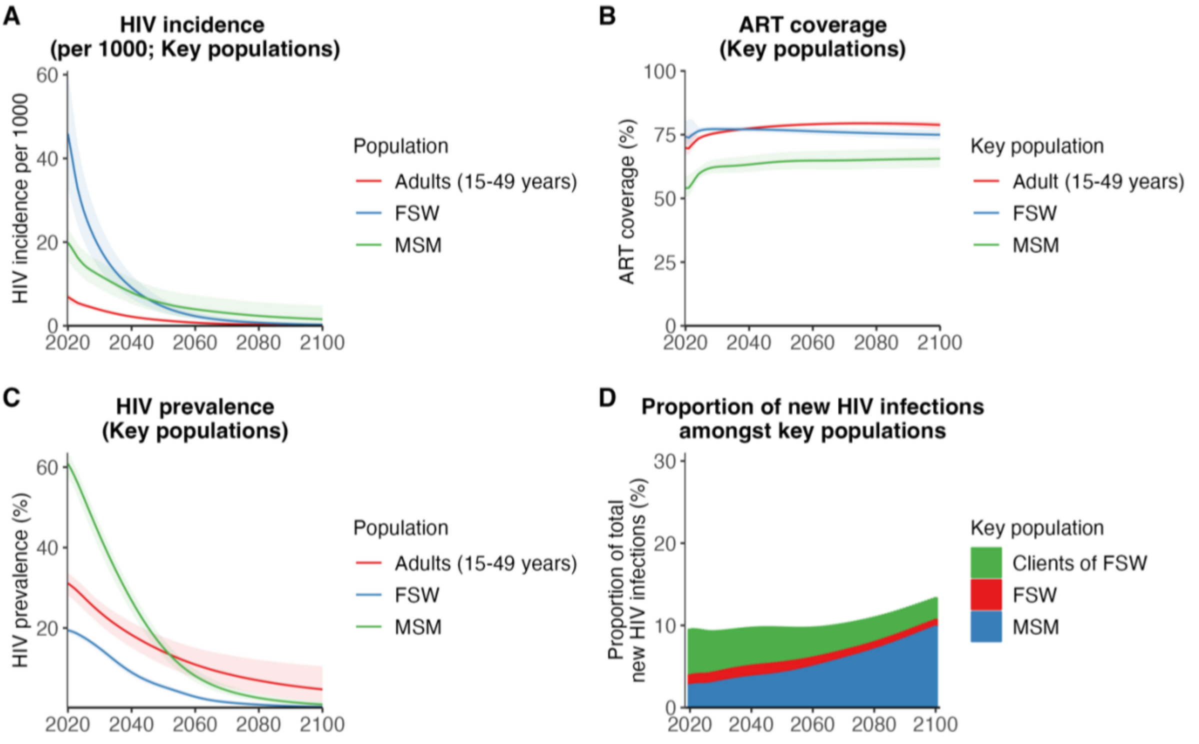

Figure S13. Status quo HIV incidence, ART coverage and contribution to total new HIV infections of key populations. (A) HIV incidence rate of adults (15–49 years), FSW (15+ years) and MSM (15+ years) per 1000, (B) ART coverage among adults (15–49 years), FSW (15+ years) and MSM (over 18+ years) and (C) proportion of total new HIV infections amongst by key populations, namely the clients of FSW (15+ years), FSW (15+ years) and, MSM (15+ years) and the clients of FSW. Figures represent status quo scenario (no general HTS reduction). Lines represent posterior mean and shaded areas are 95% confidence intervals. In (D) the shaded total area represented represents the proportion of total new HIV infections amongst key populations.

S11. References

1. Johnson LF, Dorrington RE. Thembisa version 4.5: A model for evaluating the impact of HIV/AIDS in South Africa. 2022. Accessed on: 17 Dec 2023. https://thembisa.org/content/downloadPage/Thembisa4_5report

2. Jooste S, Mabaso M, Taylor M, North A, Tadokera R, Simbayi L. Trends and determinants of ever having tested for HIV among youth and adults in South Africa from 2005–2017: Results from four repeated cross-sectional nationally representative household-based HIV prevalence, incidence, and behaviour surveys. PLOS ONE. 2020;15(5):e0232883.

3. Kaplan SR, Oosthuizen C, Stinson K, Little F, Euvrard J, Schomaker M, et al. Contemporary disengagement from antiretroviral therapy in Khayelitsha, South Africa: A cohort study. PLoS Med. 2017;14(11):e1002407.

4. Johnson LF, Meyer-Rath G, Dorrington RE, Puren A, Seathlodi T, Zuma K, et al. The effect of HIV programs in South Africa on national HIV incidence trends, 2000–2019. JAIDS Journal of Acquired Immune Deficiency Syndromes. 2022;90(2):115-23.

5. Connolly C, Simbayi LC, Shanmugam R, Nqeketo A. Male circumcision and its relationship to HIV infection in South Africa: results of a national survey in 2002. South African Medical Journal. 2008;98(10):789-94.

6. Hanscom B, Janes HE, Guarino PD, Huang Y, Brown ER, Chen YQ, et al. Brief Report: Preventing HIV-1 Infection in Women Using Oral Preexposure Prophylaxis: A Meta-analysis of Current Evidence. JAIDS Journal of Acquired Immune Deficiency Syndromes. 2016;73(5):606-8.

7. Molina J-M, Capitant C, Spire B, Pialoux G, Cotte L, Charreau I, et al. On-Demand Preexposure Prophylaxis in Men at High Risk for HIV-1 Infection. New England Journal of Medicine. 2015;373(23):2237-46.

8. McCormack S, Dunn DT, Desai M, Dolling DI, Gafos M, Gilson R, et al. Pre-exposure prophylaxis to prevent the acquisition of HIV-1 infection (PROUD): effectiveness results from the pilot phase of a pragmatic open-label randomised trial. The Lancet. 2016;387(10013):53-60.

9. Actuarial Society of South Africa. ASSA2008 AIDS and Demographic Model. 2011. Available: https://www.actuarialsociety.org.za/downloads/committee-activities/aids-models/. Accessed on 16 January 2024.

10. Meyer-Rath G, Van Rensburg C, Chiu C, Leuner R, Jamieson L, Cohen S. The per-patient costs of HIV services in South Africa: Systematic review and application in the South African HIV Investment Case. PLOS ONE. 2019;14(2):e0210497.

11. Johnson LF, van Rensburg C, Govathson C, Meyer-Rath G. Optimal HIV testing strategies for South Africa: a model-based evaluation of population-level impact and cost-effectiveness. Sci Rep 2019;9(1):12621.
